# The word order of languages predicts native speakers’ working memory

**DOI:** 10.1038/s41598-018-37654-9

**Published:** 2019-02-04

**Authors:** Federica Amici, Alex Sánchez-Amaro, Carla Sebastián-Enesco, Trix Cacchione, Matthias Allritz, Juan Salazar-Bonet, Federico Rossano

**Affiliations:** 1 Junior Research Group “Primate Kin Selection”, Max Planck Institute for Evolutionary Anthropology, Department of Primatology, Deutscher Platz 6, 04103 Leipzig, Germany; 2 University of Leipzig Faculty of Life Science, Institute of Biology, Behavioral Ecology Research Group, Talstrasse 33, 04103 Leipzig, Germany; 3 Department of Comparative and Developmental Psychology, Max Planck Institute for Evolutionary Anthropology, Deutscher Platz 6, 04103 Leipzig, Germany; 4 Department of Cognitive Science, University of California San Diego, 9500 Gilman Drive, La Jolla, CA 92093-0515 USA; 5 William James Center for Research, ISPA-Instituto Universitário, Rua Jardim do Tabaco 34, 1149-041 Lisboa, Portugal; 6 Department of Developmental and Comparative Psychology, Institute of Psychology, University of Bern, Hochschulstrasse 6, 3012 Bern, Switzerland; 7 Pedagogische Hochschule, University of Applied Sciences Northwestern Switzerland, Bahnhofstrasse 6, 5210 Windisch, Switzerland; 8 Department of International Programs, Florida State University, C/ Blanquerías 2, 46003 Valencia, Spain

## Abstract

The relationship between language and thought is controversial. One hypothesis is that language fosters habits of processing information that are retained even in non-linguistic domains. In left-branching (LB) languages, modifiers usually precede the head, and real-time sentence comprehension may more heavily rely on retaining initial information in working memory. Here we presented a battery of working memory and short-term memory tasks to adult native speakers of four LB and four right-branching (RB) languages from Africa, Asia and Europe. In working memory tasks, LB speakers were better than RB speakers at recalling initial stimuli, but worse at recalling final stimuli. Our results show that the practice of parsing sentences in specific directions due to the syntax and word order of our native language not only predicts the way we remember words, but also other non-linguistic stimuli.

## Introduction

Memory plays a central role in our lives and hundreds of studies have investigated how we store and retrieve information under different conditions^1–3^. A classic approach to the study of memory consists in presenting subjects with a list of stimuli and immediately afterwards asking them to recall as many as possible in the order they were presented. Typically, stimuli presented at the beginning (primacy items) and at the end of a list (recency items) are recalled better than stimuli from the middle^4–7^. But are these findings universal and generalizable across cultures? Most studies on memory have tested individuals that come from western, educated, industrialized, rich and democratic societies – all characteristics which are rather atypical when compared to those of other humans^8^. Moreover, the languages they speak hardly represent the linguistic diversity found across the world^9^. Yet would the language one speaks predict that person’s memory?

The relationship between language and thought is controversial. ‘Universalists’ consider differences across languages to be superficial e.g.^10^ and language to be heavily constrained by the limits of human cognition^11–13^. In contrast, ‘relativists’ contest the existence of universal properties and suggest that essential differences between languages affect the way in which speakers perceive and conceptualize the world (linguistic relativity^9,14–20^). To date, most scholars would disagree with the most radical interpretations of both approaches (i.e. a unidirectional relationship between language and thought). Indeed, recent evidence suggests, on one hand, that the language one speaks has some effect on categorization processes see for a review and, on the other hand, that learnability, and therefore the limits of our cognition, clearly affects the range of syntactic structures and semantic distinctions present among world languages^21–23^.

Even among supporters of linguistic relativity (or Whorfian hypothesis), an important distinction between a *strong* and a *weak* interpretation has been put forward^24^; see^25^. While a strong interpretation suggests that language affects cognitive *capabilities*, a weak one suggests that language is rather linked to preferred cognitive *tendencies*, in particular with respect to developing and retrieving categorical representations. Similarly, less radical interpretations of linguistic relativity suggest that language may bias attention towards certain aspects of the world^18^. This could provide interference between linguistic and non-linguistic concepts (i.e. language as interference), or more simply foster habits of processing information, which may be domain general and therefore retained even in non-linguistic domains (i.e. language as primer).

The interface between language and cognition can be detected at different levels^26^. Language, for instance, may have a clear semantic effect on thought, in that specific characteristics of languages may affect the way we conceptualize the world. Specific representations of concepts get consolidated because the language we use carves the continuum of what we perceive into specific chunks, with specific boundaries, and those constrained chunks become easier to retrieve and harder to modify. To this end, in certain perceptual/cognitive domains, the boundaries of conceptual categories strongly correlate with the semantic boundaries of the corresponding linguistic terms. A growing body of empirical work supports this view, by showing cross-linguistic differences across domains, including color^27–31^, numbers^32–36^, space^37–41^, time^25,42–44^, odor^45^ and mental states^46,47^.

Besides semantic biases, however, it is clear that repeated use of specific syntactic structures may impose specific cognitive challenges to speakers, or foster specific processing habits, which in the long term might enhance specific ways of processing information beyond the linguistic domain. Recent research on the effect of syntax on the processing of events, for instance, has shown (1) an effect of canonical noun-adjective word order on the speed at which noun categories are retrieved^48^, (2) and on recognition memory and similarity judgments while classifying items^49^, as well as (3) an effect of transitive vs. intransitive structures, and agentive vs. non-agentive structures (such as “she broke the vase” vs. “the vase broke itself”), on people’s capacity to remember who was the agent in accidental events^50–52^. To our knowledge, however, there is surprisingly no research on the effects of the syntax of one’s native language on cognitive processes unrelated to categorization and discrimination tasks.

Here we test the linguistic relativity hypothesis, along with the previously established perspective that the main effect of language on thought is likely due to habituation in terms of strategies deployed to perceive, interpret and remember the world that surrounds us^53^. We believe to be the first ones to focus not on the semantic or syntactic effect of language on cognitive representations, but rather on the effect of syntax on the cognitive processes through which people recall information. Specifically, we investigate whether memory retrieval in both linguistic and non-linguistic tasks is predicted by the way languages normatively order words within sentences. Languages vary substantially in their branching direction, that is, the order in which the nucleus/head and dependent/modifier linguistic units are usually presented in a sentence. In typical right-branching (RB) languages, like Italian, the head of the sentence usually comes first, followed by a sequence of modifiers that provide additional information about the head, creating parse trees that grow down and to the right: the head noun typically precedes genitive noun phrases (e.g. “mother of John”) or relative clauses (e.g. “the man who was sitting at the bus stop”), for instance. In contrast, in left-branching (LB) languages, like Japanese, modifiers generally precede heads (e.g. “John’s mother” and “who was sitting at the bus stop, the man”), creating parse trees that grow down and to the left. In addition to specific ordering within phrases, languages differ also in terms of the positions of subject, verb and object within a clause. The two most common distributions, accounting for more than 4/5 of all natural languages spoken in the world see^54^, are Subject-Verb-Object (SVO) and Subject-Object-Verb (SOV). It has long been noted that languages with SVO (e.g. Italian) tend to use prepositions and therefore put modifiers after the head (i.e. tend to be RB), while SOV languages (e.g. Japanese) tend to prefer postposition and place modifiers before the head (i.e. tend to be LB) see e.g.^55,56^. One of the hypotheses behind this correlation is that languages tend to be consistently LB or consistently RB to facilitate language processing e.g.^55,57^.

To date, there is no consensus on how LB and RB structures are parsed. In RB languages, speakers could process information incrementally with a low risk of re-analysis, given that heads are presented first and modifiers rarely affect previous parsing decisions. Although final modifiers surely refer to initial heads in RB languages, initial heads are clear from the very beginning and independently of the final modifiers, which only add information to the heads. In contrast, LB structures can be highly ambiguous until the end, because modifiers, that usually come first, often acquire a clear meaning only after the head has been parsed see^58,59^. Therefore, LB speakers may need to consistently delay parsing decisions to avoid extensive backtracking, retaining initial modifiers in working memory until the head is encountered or the verb is produced, and the sentence can be given a meaning. In contrast, RB speakers may make parsing decisions immediately, and thus they would require no especially enhanced memory for the initial information while parsing. In line with this, some studies suggest that LB speakers may more easily parse double-embedded relative clauses, as compared to RB speakers, also because of a higher WM capacity e.g.^60–62^.

In natural conversation, all natural languages are processed fast and efficiently, and successfully deployed in fast and timely turn-taking during social interaction^63^, probably because language comprehension is facilitated by other contextual factors, such as current topic of conversation, recent referential mentioning, salience and priming effects^64^. Nonetheless, there is evidence that the branching direction of ones’ own language may play a role in parsing information. A bias towards the branching of one’s native language emerges early in life^59,65^, and even young children generalize it when learning a second language^66^. Speakers seem to develop a “bias” toward the branching direction more common in their language, so that LB structures are harder to process for RB speakers (due to the higher working memory needed to retain the intermediate products of computation^67–69^; see^70^ for experimental evidence), but they are more accessible than RB structures for LB speakers^65,71,72^. Therefore, LB speakers might rely more on strategies other than word order to resolve ambiguity during sentence processing^59^. For instance, they may also rely on statistical information about the relative frequencies with which different syntactic structures and other linguistic material occur in the language^73–75^; see^76,77^ for a discussion of processing in LB languages. Thus, processing difficulty would simply increase when the input does not match expectations^77,78^, but would remain as low as expected otherwise.

Accordingly, languages tend to be consistently RB or LB^55^, because consistently sticking to just one parsing strategy may reduce the processing difficulties associated with a mixture of RB and LB structures^9,55,57^. This sensitivity to the branching direction of a language may be cognitively so relevant to also affect the way in which humans remember and/or process sequences of stimuli. Therefore, speakers from languages that vary in their branching may differ in the way they process and/or remember not only words, but also other non-linguistic stimuli. More specifically, we expected LB speakers to better recall initial stimuli as compared to RB speakers, as real-time sentence comprehension relies more heavily on retaining initial information in LB languages.

In order to test this hypothesis, we selected four RB languages (Ndonga, Khmer, Thai, Italian) and four LB languages (Sidaama, Khoekhoe, Korean, Japanese), using the World Atlas of Language Structures (WALS^79^). To determine the degree of branching in each language, we used the following word order criteria: order of object-verb, genitive-noun, relative clause-noun, and clause-subordinate. All languages were consistently RB or LB according to all these criteria (except for Sidaama, for which the clause-subordinate order is not classified as either consistently RB or LB by the WALS). In comparison, English is consistently RB for three out of four of these criteria. For each language, we tested 24–30 adult native speakers of both sexes, in three widely used working memory (WM) and three widely used short-term memory (STM) tasks, containing sets of 2–9 numerical, spatial or word stimuli (see Methods). These tasks are well-established span tasks which have been implemented in the Attention & Working Memory Lab by Engle’s research group and validated across a variety of studies see^80,81^. Ambiguity exists on the relationship between these two distinct but highly correlated constructs, but most cognitive psychologists would agree that while STM is a storage component of no longer externally available information, WM also contains an attention component aimed at maintaining memory representations in the face of concurrent processing, distraction and attention shifts e.g.^82–85^, and has an active role in language e.g.^86,87^. Indeed, several studies have demonstrated the influence of WM on sentence processing^88,89^; see^90^, with WM tasks correlating much better with sentence comprehension as compared to STM tasks e.g.^91,92^; see^87^. Therefore, we expected branching to predict individuals’ ability to recall stimuli in WM but not in STM tasks.

In our study, subjects had to sequentially recall the stimuli right after each presentation. To explore whether branching predicted individuals’ ability to better recall initial (primary) or final (recency) stimuli, for each participant we coded the number of correct items recalled in the first half and in the last half of each set of stimuli (the middle stimulus was not coded in lists with odd numbers). The stimuli position (initial, final) was then included as test predictor - together with stimuli kind (spatial, numerical, word) and branching direction (left, right) - in two different models, one for STM tasks and the other for WM tasks, while controlling for repeated observations, multiple components of socio-economic status and individual demographic variables (see Method for a detailed description). This ensured that differences in performance across linguistic groups depended on the position of the recalled stimuli, while controlling for several other factors.

## Methods

### Participants

For each linguistic group, we recruited 30 native speakers (with the exception of South Korea, where only 25 participants were tested due to logistic problems). Participants were of both sexes, aged between 14 and 43. They resided either in a village/town (i.e. <100.000 inhabitants) or in a city (i.e. >100.000 inhabitants), and had a different number of siblings (from 0 to 16). Participants differed in their education level, had different occupations and monthly income. Participants further varied in the second languages they spoke and in their level of proficiency. English was the most common second language spoken in all linguistic groups, with the exception of Khoekhoe (who mostly spoke Afrikaans as a second language) and Sidaama (who mostly spoke Amharic as a second language). For more details, see Table 1 and Supplementary Information.

**Table 1:** Information on the subjects included in the analyses.

Branching Direction	Linguistic Group	Country	Number Subjects	Sex: Females - Males	Age^a^: Mean (Range)	% City^b^	Number Siblings: Mean (Range)	Education Level^c^: Mean (Range)	Occupation^d^	Monthly Income (€): Mean (range)	Most Spoken 2^nd^ Language	Knowledge Opposite Branching^e^
Right	Italian	Italy	30	11–19	37 (15–40)	100	1 (0–2)	14 (9–15)	2–0–3–1–22–2	1780 (0–3500)	English	0
	Khmer	Cambodia	30	20–10	25 (15–43)	0	3.8 (1–7)	9 (3–12)	11–3–6–2–0–8	41 (0–186)	English	0
	Oshiwambo	Namibia	30	20–10	25 (15–40)	6.7	4.3 (0–11)	7 (2–12)	10-2-2-7-2-7	151 (0–874)	English	0
	Northern Thai	Thailand	30	16–14	28 (15–40)	6.9	1.9 (0–6)	12 (6–17)	0-0-8-6-5-11	171 (0–389)	English	0
				**67–53**	**29** (**15–43**)	**28**.**6**	**2**.**7** (**0–11**)	**10** (**2–17**)	**23-5-19- 16-29-28**	**536** (**0–3500**)	**English**	**0**
Left	Japanese	Japan	30	16–14	21 (19–24)	100	1.5 (0–3)	15	0-0-0-0-0-30	0	English	1.6 (1–2)
	Korean	South Korea	24	14–10	22 (18–30)	87.5	1.3 (0–2)	15 (13–15)	0-0-0-1-0-23	0	English	1.5 (1–2)
	Khoekhoe	Namibia	29	21–8	25 (14–40)	13.8	4.9 (0–16)	7 (0–15)	7-2-3-5-0-12	156 (0–1036)	Afrikaans	1.4 (1–2)
	Sidaama	Ethiopia	30	10–20	23 (16–35)	76.7	7.2 (0–14)	9 (0–15)	5-2-6-3-0-14	18 (0–134)	Amharic	0.2 (0–2)
				**61–52**	**23** (**14–40**)	**69**.**0**	**3**.**8** (**0–16**)	**11** (**0–15**)	**12-4-9- 9-0-79**	**45** (**0–1036**)	**English**	**1**.**2** (**0–2**)

^a^Age is in years.

^b^Percentage of people living in the city.

^c^Number of years of formal education.

^d^Number of participants being occupied in one of the following categories: unemployed participants; participants working in the primary sector; in the secondary sector; in the tertiary sector (commerce or tourism); in the tertiary sector (other services); students.

^e^Degree of knowledge of second languages with a branching opposite to the native language (from 0 to 2, according to a simplified version of the ILR scale).

All experimental procedures had been approved by the ethical committee at the University of Bern, Switzerland (2016-06-00006), all experiments were performed in accordance with European guidelines and regulations, and informed consent was obtained from all participants.

### Experimental protocol

Testing took place in surroundings that were familiar to the participants, such as schools, community centers and private homes. Individuals were generally tested alone, unless they felt uncomfortable and asked for other people being present, in which case these were sat at a certain distance behind the computer screen and instructed not to interfere in any way with the testing procedure. For each population, one research assistant collected the data together with a local research assistant translating the procedure, when needed (i.e. in Cambodia, Ethiopia, Japan, Korea and Namibia). In Italy and Thailand no local research assistant was needed, as the research assistant collecting the data was a native speaker of the language tested. Overall, a native speaker of the local language conducted recruiting, consenting and testing for all populations tested. Written consent was obtained before testing, while biographical information was obtained at the end of the tasks, by noting participants’ name, sex and age, residence, number of siblings, main occupation, approximate monthly income, educational level, native language and proficiency in other languages.

Each participant was tested in 6 different memory tasks, administered one after the other on a laptop, with approximately one-minute breaks in-between. The six tasks were three short-term memory (STM) tasks with words as stimuli (WS = word span), with numbers as stimuli (DS = digit span), or with spatial stimuli (MS = matrix span); and three working memory (WM) tasks with words as stimuli (OS = operation span), with numbers as stimuli (CS = counting span), or with spatial stimuli (SS = symmetry span). For these tasks, we adapted the classic automated span tasks programmed with E-prime and implemented in the Attention & Working Memory Lab by Engle’s research group^80,81^. All tasks have been validated across a variety of studies and basically test STM and WM by requiring individuals to observe a series of stimuli and recall them immediately afterwards, in the same order they were presented. Before each task started, participants were instructed about the procedure and provided with two examples containing two stimuli. Moreover, they were also reminded that stimuli had to be sequentially recalled, in the same order as they were presented. In case the procedure was not clear, it was explained again until the participant understood it. Throughout the tasks, the experimenter made no suggestions, but could motivate participants regardless of their performance by reassuring them that they were doing fine. The order of tasks was pseudo-randomized and counterbalanced across subjects, but the order of stimuli and trials within each task was the same for all participants (see Supplementary Information for more details).

### STM tasks

In the STM-WS task, participants were presented with 18 test trials, each one containing 2–7 stimuli. The stimuli consisted of 600_px_ × 800_px_ pictures with images of common animals and objects (e.g. a cat, a hen, a leaf, an ant, a cloth), being visible for 2000 ms in the middle of the screen. Before the task started, individuals were instructed to observe the series of pictures on the screen, name each of them aloud as soon as it appeared, and recall them aloud in the same order they had appeared, as soon as question marks appeared on the screen. The experimenter audio-recorded all trials.

In the STM-DS task, participants were presented with 21 test trials containing 3–9 stimuli. The stimuli consisted of numbers from 1 to 9 (presented as 100_px_ × 150_px_ images with a black number on a white background), which were visible for 2000 ms in the middle of the screen. Before the task started, individuals were instructed to observe the series of numbers on the screen and then recall them in the same order they had appeared, as in the previous task. Participants provided their response on coding sheets with series of 9 squares, so that each square could contain one number.

In the STM-MS task, participants were presented with 18 test trials containing 2–7 stimuli. The stimuli consisted of 4 × 4 squared matrixes (presented as 400_px_ × 300_px_ images) with a black grid on a white background, and one of the 16 squares inside being colored red in each stimulus (the position of this red square was different depending on the stimulus). Each stimulus was visible for 2000 ms in the middle of the screen. Before the task started, individuals were instructed to observe the series of matrixes on the screen and then recall the position of each red square in the same order they had appeared, by writing them down in a coding sheet as soon as questions marks appeared on the screen.

### WM tasks

In the WM-OS task, participants were presented with 12 test trials containing 2–5 stimuli. The stimuli consisted of 600_px_ × 800_px_ pictures with images of common animals and objects (as in the STM-WS task), and three little squares with a variable number of red dots inside, which served as stimuli for the distracting task. Before the task started, individuals were instructed to observe the series of pictures on the screen, name each of them aloud as soon as it appeared, solve the distracting task (by subtracting the red dots in a box from the red dots in the other one, and telling aloud whether the result corresponded to the number of red dots in the third box; i.e. distracting task), and then recall the name of the pictures aloud in the same order they had appeared, as soon as question marks appeared on the screen. In this task, each stimulus remained in the middle of the screen until it was named and the mathematical operation was solved. The experimenter audio-recorded all trials.

In the WM-CS task, participants were presented with 15 test trials containing 2–6 stimuli. The stimuli consisted of 600_px_ × 800_px_ pictures with a grey background and a varying number of blue circles, blue squares and green circles (with the number of blue circles in each image varying from 3 to 9). Before the task started, individuals were instructed to observe the series of images on the screen, count aloud the number of blue circles among other figures in each image (i.e. distracting task), repeat this number aloud and then recall aloud the series of final numbers in the same order they had appeared, as soon as question marks appeared on the screen. Each stimulus remained in the middle of the screen until the blue circles had been counted. The experimenter audio-recorded all trials.

In the WM-SS task, participants were presented with 12 test trials containing 2–5 stimuli. The stimuli consisted of 4 × 4 squared matrixes (presented as 400_px_ × 300_px_ images) with a black grid on a white background (as in the STM-MS task), and one of the 16 squares inside being colored red in each stimulus. These matrixes were alternated to 8 × 8 squared matrixes of the same size, serving as stimuli for the distracting task: some of the 64 squares were colored black, forming a muster that could either be symmetrical or asymmetrical along the vertical axis. Before the task started, individuals were instructed to observe the series of 4 × 4 matrixes on the screen, assess aloud whether the 8 × 8 symmetry matrixes were symmetrical or not (i.e. distracting task), and then recall the position of each red square in the 4 × 4 matrixes in the same order they had appeared, by writing them down in a coding sheet as soon as the question marks appeared on the screen. All matrixes were visible for 2 seconds in the middle of the screen, but 4 × 4 matrixes were only visible after the previous symmetry judgment had been done. On a piece of paper, the experimenter further noted the participants’ responses to the distracting task.

### Scoring

We transcribed all participants’ responses from the audios and coding sheets. We then compared the recalled stimuli to the stimuli as named during the stimuli presentation. For each trial, we divided the list of stimuli presented in two halves and separately coded the number of correct responses for the first half (i.e. initial stimuli) and for the second half (i.e. final stimuli). For the first half, we coded whether the first stimulus recalled corresponded to the first stimulus having been presented, whether the second stimulus recalled corresponded to the second stimulus having been presented, and so on. For the second half, we coded whether the last stimulus recalled corresponded to the last stimulus having been presented, the second to last stimulus recalled corresponded to the second to last stimulus having been presented, and so on. Crucially, coding the final stimuli starting from the end ensured that mistakes in recalling initial stimuli did not affect the response for the final stimuli, as a correct response required that both identity and order of stimuli were recalled correctly.

### Inter-observer reliability

A second observer recoded 11.6% of all the trials and inter-observer reliability was excellent (for the sum of correct initial stimuli in each trial: Cohen’s k = 0.955, N = 2592, *p* < 0.001; for the sum of correct final stimuli in each trial: Cohen’s k = 0.940, N = 2592, *p* < 0.001).

### Statistical analyses

Before conducting the analyses, we excluded some participants from the sample. In particular, although all participants alleged to be native speakers of the language they were going to be tested for, based on the interactions with the participants we inferred that one Korean and one Khoekhoe-speaker were not native speakers of those languages and we therefore dropped them from the analyses. We further excluded from the analyses one Sidaama who failed to count the blue circles aloud in the distracting task of the WM-CS task (as the distracting task was not implemented, transforming the nature of the WM task). Finally, we excluded 68 trials (i.e. 0.3% of the remaining trials), due to problems with the audio-recordings, participant’s failure to understand the procedure, participant’s distraction or others’ interference in the task.

All analyses were conducted using generalized linear mixed models (GLMM)^93^ and were run using R statistics (version 3.2.3) with the lme4 package^94^. We ran one model for the WM tasks, and one for the STM tasks, both with a Poisson structure. In the models, we included participants’ performance for initial and final stimuli in each trial of the WM numerical, spatial and word tasks (*N* = 18050), and in each trial of the STM numerical, spatial and word tasks (*N* = 26470), respectively. All numerical variables were z-transformed, to obtain comparable and more easily interpretable coefficients^95^. To analyze the effect of test predictors (i.e. the predictors of interest) on the response, we compared each full model (including both control and test predictors) to a corresponding null model (only including control predictors). When test predictors have a significant effect on the response, the full-null model comparison is significant. To obtain the *p* values for the individual fixed-effects we conducted likelihood-ratio tests^96^. In order to rule out collinearity, we checked variance inflation factors (VIF)^97^ and overall VIF values were generally close to one (maximum VIF = 3.26). All models were stable.

In both models, the dependent variable was the number of correct stimuli identified (initial and final). Moreover, in both models, we included three test predictors: branching direction (right or left), kind of stimuli (numerical, spatial and word), and stimuli position (initial or final), as well as their 2- and 3-way interactions. Main branching direction based on (i) the SVO/SOV order, (ii) the presence of head nouns preceding/following (iii) genitive and (iv) relative clauses, and (v) separate adverbial subordinators at the beginning/end of subordinate clauses^79^. See Supplementary Information for more details.

As control predictors we included (i) fixed effects known to potentially affect WM and/or STM, crucially including all possible random slopes, and (ii) random effects. In this way, we could (i) assess the effect of our test predictors after controlling for the effect of other potentially confounding variables, and (ii) account for the non-independence of data points. As fixed effect variables we included: participant’s sex (2 levels e.g.^98–100^), participant’s age (from 14 to 43 years old e.g.^101–103^), number of siblings (from 0 to 16 see^104^), residence (village/town or city, with threshold set at 100.000 inhabitants; as living in cities may favour enhanced spatial memory), level of education (depending on the years spent at school/university e.g.^105,106^), occupation (unemployed, working in the primary sector, in the secondary sector, in commerce or tourism, in other areas of the tertiary sector, students e.g.^107–109^), centered income (as the deviation of each participant’s monthly income from the average national income e.g.^110,111^), average national income (from 58 to 2588 €, as calculated by the International Labour Organization), knowledge of a language with an opposite branching (none, low to middle, middle to high, as based on a simplified version of the Interagency Language Roundtable scale for Language Proficiency by the U.S. Department of State; as this could reduce the effect of the native branching), number of stimuli in each trial (from 2 to 9 see e.g.^80^), trial number within each task (from 1 to 21), and (only in the WM tasks) the percentage of correct choices in the distracting trials. Note that the inclusion of all these fixed effects makes our results especially robust, as they assess the effect of test predictors (which are *a priori* defined), independently of other potential confounding factors, also defined *a priori*. As random effect variables, we included language, participant’s identity and trial identity (given that each trial was coded twice: the first half starting from the beginning, and the second half starting from the end), to account for the non-independence of data points.

## Results

### The effects of branching on WM

**Figure 1:**
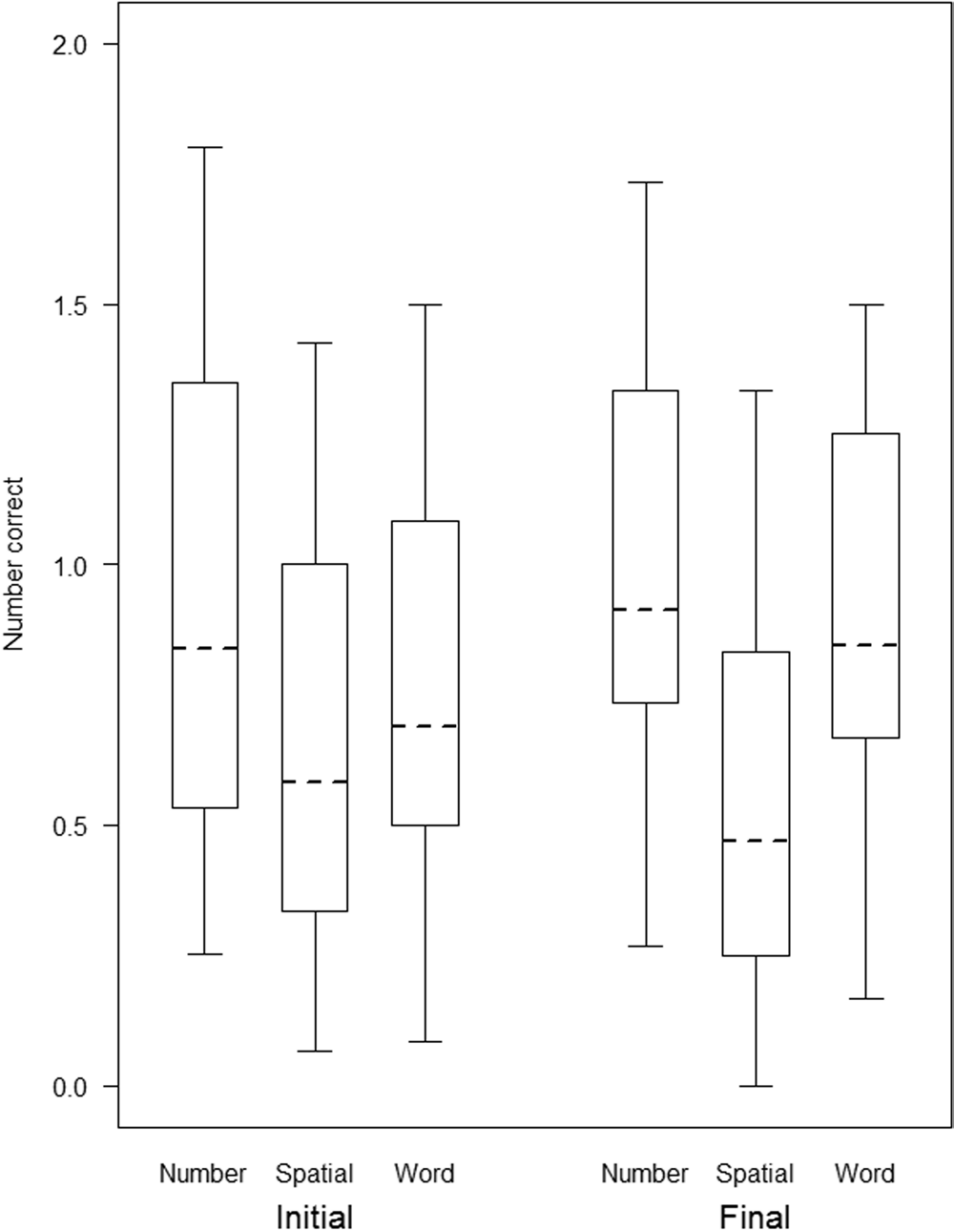
Box-plot representing the data distribution for the number of correct initial and final stimuli in WM tasks with numeric, spatial and word stimuli from a generalized linear mixed model (GLMM). The horizontal ends of the box represent the 75% and 25% quartiles, and the ends of the whiskers represent the 97.5% and 2.5% quartiles respectively. The dotted line represents the model estimates.

**Figure 2:**
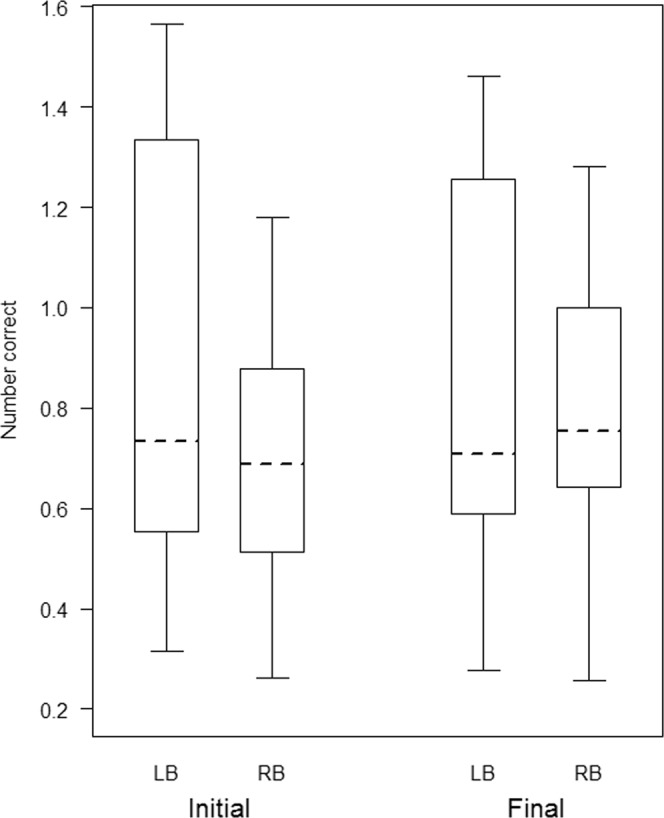
Box-plot representing the data distribution for the number of correct initial and final stimuli in WM tasks for left-branching (LB) and right-branching (RB) participants from a generalized linear mixed model (GLMM). The horizontal ends of the box represent the 75% and 25% quartiles, and the ends of the whiskers represent the 97.5% and 2.5% quartiles respectively. The dotted line represents the model estimates.

**Figure 3:**
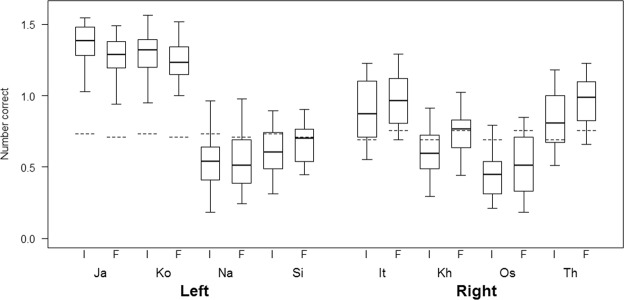
Box-plot representing the data distribution for the number of correct initial (I) and final (F) stimuli in WM tasks for left-branching (LB) participants of each language (Ja = Japanese, Ko = Korean, Na = Khoekhoe, Si = Sidaama), and for right-branching (RB) participants of each language (It = Italian, Kh = Khmer, Os = Oshiwambo, Th = Northern Thai), from a generalized linear mixed model (GLMM). The horizontal ends of the box represent the 75% and 25% quartiles, and the ends of the whiskers represent the 97.5% and 2.5% quartiles respectively. The dotted line represents the model estimates.

The comparison between the full model and the null model was significant (GLMM: *p* < 0.001, N = 18050, ${\chi }_{11}^{2}$ = 55.81). After dropping the non-significant three-way interaction from the model (branching*kind of stimuli*position of stimuli) (GLMM: *p* = 0.65, N = 18050, ${\chi }_{2}^{2}$ = 0.86), we found two significant two-way interactions. A first interaction between kind of stimuli and stimuli position revealed that participants were better at recalling number and word stimuli in the final position, but worse at recalling spatial stimuli in the final position (GLMM: *p* < 0.001, N = 18050, ${\chi }_{2}^{2}$ = 23.45; Fig. 1). Crucially, a second interaction between branching and stimuli position revealed that LB participants were better than RB participants at recalling initial stimuli, but worse at recalling final stimuli (GLMM: *p* = 0.01, N = 18050, ${\chi }_{1}^{2}$ = 6.48; Fig. 2). Consistent with the aggregate data, Fig. 3 shows that in all RB languages, participants were better at recalling final as compared to initial stimuli, and in all LB languages (with the exception of Sidaama) participants were better at recalling initial as compared to final stimuli.

### The effects of branching on STM

**Figure 4:**
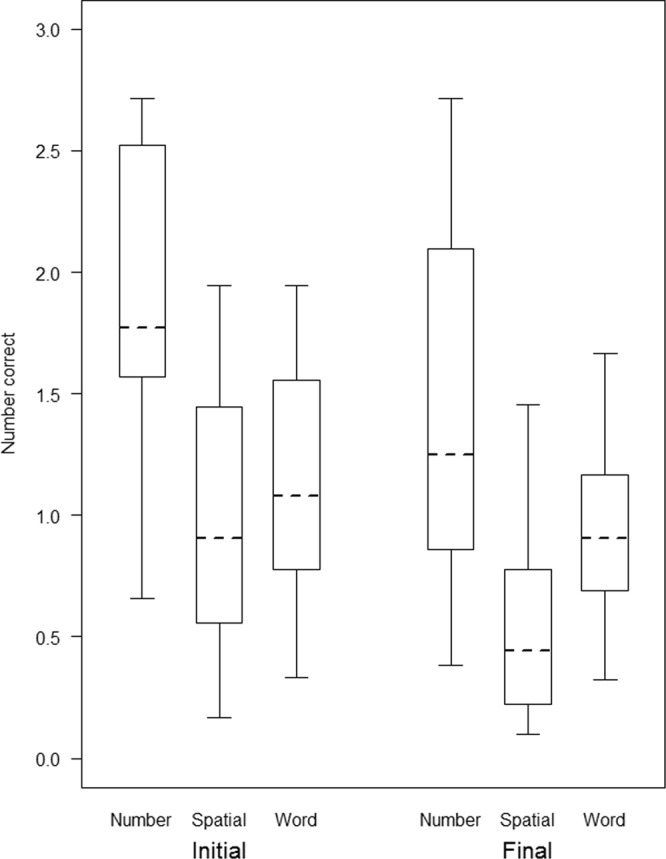
Box-plot representing the data distribution for the number of correct initial and final stimuli in STM tasks with numeric, spatial and word stimuli from a generalized linear mixed model (GLMM). The horizontal ends of the box represent the 75% and 25% quartiles, and the ends of the whiskers represent the 97.5% and 2.5% quartile respectively. The dotted line represents the model estimates.

The comparison between the full model and the null model was significant (GLMM: *p* < 0.001, N = 26470, ${\chi }_{11}^{2}$ = 78.79). After dropping the non-significant three-way interaction (branching*kind of stimuli*position of stimuli) (GLMM: *p* = 0.19, N = 26470, ${\chi }_{2}^{2}$ = 3.23), we found a significant two-way interaction between kind of stimuli and position of stimuli. In particular, participants were overall better at recalling initial stimuli as compared to final stimuli in all tasks, and this effect was steeper in number stimuli compared to spatial and word stimuli (GLMM: *p* < 0.001, N = 26470, ${\chi }_{2}^{2}$ = 27.42; Fig. 4). In contrast, no effect of branching was found, indicating that LB and RB speakers did not differ in their performance in STM tasks (GLMM: *p* = 0.78, N = 26470, ${\chi }_{1}^{2}$ = 0.076).

## Discussion

As predicted, LB and RB speakers were significantly different in their ability to recall initial and final stimuli, showing a clear link between branching direction and working memory (WM). In WM tasks, LB participants were better than RB participants at recalling initial stimuli (and RB were better at recalling final stimuli), and this pattern held for each language separately (with the exception of Sidaama). These results confirm our hypothesis and suggest that sensitivity to branching direction predicts the way in which humans remember and/or process sequences of stimuli, as real-time sentence comprehension relies more heavily on retaining initial information in LB languages but not in RB languages.

Sidaama was the only language failing to follow this pattern, but there are at least two reasons as to why this might be the case. Firstly, all languages were consistently RB or LB according to four word order criteria (see Methods), with the exception of Sidaama, for which the clause-subordinate order follows no consistent branching direction^79^. Secondly, the Sidaama participants that we tested were the most secluded group compared to all other populations tested, and in contrast to the other tested groups they had had little to no previous contact with technologies (including laptops and audio-recorders). This resulted in WM trials lasting significantly longer than in the other groups, with earlier stimuli becoming comparatively less accessible, and this likely explains the difference between performance by the Sidaama and the other LB participants (Mean session length ± SE for Sidaama: 9.5 minutes ± 3.2, Khoekhoe: 5 ± 1; Korean: 6.3 ± 1.0; Japanese: 6.4 ± 1.0).

Japanese- and Korean-speakers’ performance was impressive for both initial and final stimuli, although initial stimuli were better recalled than final ones (Fig. 3). These results are not surprising, as Japanese- and Korean-speakers were mostly students, with a much higher familiarity with being tested on computers than most other participants. Such a higher familiarity likely resulted in overall better performance see e.g.^112^, although there is no reason to assume that it provided them with a special advantage to remember initial versus final stimuli. Moreover, in our models we explicitly controlled for participants’ occupation (and for several other factors differing across participants and groups, which might have affected their performance, see Methods), suggesting that these differences cannot explain the results obtained.

As predicted, the effect of branching direction was confined to performance in WM tasks, while RB and LB speakers did not differ in their STM performance. One plausible explanation is that only WM has an active role in language and sentence processing e.g.^86,87,90,113,114^. Moreover, while WM tasks largely reflect a domain-general factor, STM tasks tend to be much more domain specific^115^. Therefore, the effect of language on non-linguistic cognition might be more limited in STM tasks. Finally, it is also possible that the effect of branching direction on performance in WM (but not STM) tasks depends on output interference (i.e. degradation of later-recalled items in the list. due to the interference of initially recalled items) being stronger in STM than WM tasks^116^, and thus wiping out the branching effect in STM tasks, where initial stimuli were recalled much better than final ones (Fig. 4).

In contrast, the link between branching and performance in WM tasks held regardless of the stimuli used (i.e. word, numerical or spatial stimuli). This may be surprising, because branching direction may be expected to more likely predict performance in verbal rather than spatial WM tasks, as only the former selectively tap capacities which are essential for sentence processing. However, although spatial and verbal memory are usually considered two different WM components, it is to date unclear how easily transfers take place between these different components. Transfer from WM training in the lab, for instance, is generally limited see^112^, but there is evidence that interventions improving verbal WM may also have benefits that transfer to spatial WM e.g.^117^. Moreover, it is interesting to note that several participants across different linguistic groups (both RB and LB) spontaneously reported, at the end of the tasks, to have coded spatial information on the grid as numerical information: instead of visualizing and later recalling the spatial position of the red square in WM spatial tasks, they reported to have attributed sequential numbers to the squares on the grid, so that the number corresponding to the red square was kept in memory and later recalled. This approach may have transformed a classic spatial task into a more verbal one, which may be more likely subject to branching effects.

Taken together, our results suggest that the link between language and thought might not be just confined to conceptual representations and semantic biases, but rather extend to syntactic structures and the very sequential processing of information. Specific characteristics of a language appear to predict not only the way we perceive and conceptualize the world see^9^, but also the way we process, store and retrieve information. This is especially relevant, as the ability to maintain sequential information in working memory is crucial for a wide range of higher cognitive functions, including reading, problem-solving, decision-making and planning^2,80,81,85,110,112,118^. Therefore, the need to parse sentences in a specific direction, day by day, might affect our way to remember words and other stimuli also in a non-linguistic context. This is in line with previous findings, showing that extensive experience, like biologically relevant behaviors engaging higher cognitive functions (e.g. extensive learning, playing music), can drastically affect our memory and even cause long-term structural changes to our brain, well into adulthood^119–121^.

In future work, the inclusion of languages with mixed branching and free word order, while controlling for the frequency of non-canonical word order in each language, would likely provide valuable further insights into the exact link between branching and memory. Free word order languages, in particular, seem to provide an especially interesting test for the linguistic relativity hypotheses: sentences containing the same words in a different order, for instance, appear to be considered repetitions by speakers of free word order languages^122^. The fact that branching and word order may be linked to such a fundamental cognitive process like memory opens up new exciting avenues for psycholinguistic research towards expanding the pool of languages and populations investigated. With more than 7000 languages in the world, we have a uniquely rich pool to study the relation between language and cognition. Preserving and investigating the wealth of this diversity is not only ethical, but also scientifically crucial to ultimately address the age-old question concerning the relation between language and thought.

## Supplementary information

Supplementary Material

Dataset 1

## Data Availability

Data are available as Supplementary Material.
