# Supplementary material for: The word order of languages predicts native speakers’ working memory
Source: Sci Rep. 2019 Feb 4;9:1124. doi: 10.1038/s41598-018-37654-9 (PMC6362290; doi:10.1038/s41598-018-37654-9)
Supplement: Supplementary file 1 — Supplementary Material [file 41598_2018_37654_MOESM1_ESM.docx]

TITLE: The word order of languages predicts native speakers’ working memory

AUTHOR NAMES: Federica Amici^1,2, *^, Alex Sánchez-Amaro^3, 4^, Carla Sebastián-Enesco^5^, Trix Cacchione^6,7^, Matthias Allritz^3^, Juan Salazar-Bonet^8^, Federico Rossano^4^

AUTHOR AFFILIATIONS:

^1^ Junior Research Group "Primate Kin Selection", Max Planck Institute for Evolutionary Anthropology, Department of Primatology, Deutscher Platz 6, 04103 Leipzig, Germany

^2^ University of Leipzig Faculty of Life Science, Institute of Biology, Behavioral Ecology Research Group, Talstrasse 33, 04103 Leipzig, Germany

^3^ Department of Comparative and Developmental Psychology, Max Planck Institute for Evolutionary Anthropology, Deutscher Platz 6, 04103, Leipzig, Germany

^4^ Department of Cognitive Science, University of California San Diego, 9500 Gilman Drive, La Jolla, CA 92093-0515, USA

^5^ William James Center for Research, ISPA-Instituto Universitário, Rua Jardim do Tabaco 34, 1149-041, Lisboa, Portugal

^6^ Department of Developmental and Comparative Psychology, Institute of Psychology, University of Bern, Hochschulstrasse 6, 3012, Bern, Switzerland

^7^ Pedagogische Hochschule, University of Applied Sciences Northwestern Switzerland, Bahnhofstrasse 6, 5210, Windisch, Switzerland

^8^ Department of International Programs, Florida State University. C/ Blanquerías 2, 46003, Valencia, Spain

**SUPPLEMENTARY INFORMATION**

**Table of contents**

1. **Participants………………………………………………………………………..2**

**2. Site and language information……………………………………………………4**

2.1. Cambodia (Khmer) ………………………………………………………4

2.2. Ethiopia (Sidaama) ………………………………………………………4

2.3. Italy (Italian)……………………………………………………………...5

2.4. Japan (Japanese)………………………………………………………….6

2.5. South Korea (Korean)…………………………………………………….7

2.6. Namibia (Nama)………………………………………………………….8

2.7. Namibia (Oshiwambo)……………………………………………………8

2.8. Thailand (Northern Thai)…………………………………………………9

**3. Experimental protocol……………………………………………………………10**

3.1. STM-WS task: short-term memory with word stimuli…………………..11

3.2. STM-DS task: short-term memory with numerical stimuli……………...13

3.3. STM-MS task: short-term memory with spatial stimuli…………………13

3.4. WM-OS task: working memory with word stimuli………………………14

3.5. WM-CS task: working memory with numerical stimuli…………………15

3.6. WM-SS task: working memory with spatial stimuli……………………..16

3.7. Questionnaire……………………………………………………………..17

3.8. Modification of stimuli between sites……………………………………18

**4. Inter-observer reliability………………………………………………………….18**

**5. Model analyses…………………………………………………………………….18**

5.1. Test predictors for STM and WM models……………………………..…19

5.2. Control predictors: Fixed effect variables for all model…….….………..19

5.3. Control predictors: random effect variables for all models……..………..21

5.4. Model 1: the WM model..…………………………………………………21

5.5. Model 2: the STM model………………………………………………….22

**6. References……………………………………………………….,………………….26**

**1. Participants**

For each linguistic group, we recruited 30 native speakers (with the exception of South Korea, where only 25 participants were tested due to logistic problems). All subjects alleged to be native speakers of a single language. However, based on the interactions with the participants, we inferred that one Korean and one Nama-speaker were not native speakers of those languages and we therefore dropped them from further statistical analyses (see Table S1).

Participants were of both sexes, aged between 14 and 43. They resided either in a village/town (i.e. <100.000 inhabitants) or in a city (i.e. >100.000 inhabitants), and had a different number of siblings (from 0 to 16). Participants differed in their education level, ranging from 0 to 17 years of formal education. Moreover, we included participants with different occupations. Depending on their main occupation, participants were classified as unemployed participants, participants working in the primary sector, in the secondary sector, in the tertiary sector (commerce or tourism), in the tertiary sector (other services), or students. Participants also had a different monthly income, ranging from 0 to approximately 3500 euros a month.

Finally, participants varied in the second languages they spoke and in their level of proficiency. English was the most common second language spoken in all linguistic groups, with the exception of Nama (who mostly spoke Afrikaans as a second language) and Sidaama (who mostly spoke Amharic as a second language). For more details on how the knowledge of second languages was taken into account in this study, see below (5.2).

**Table S1**. Information on the subjects included in the analyses

| BRANCHING DIRECTION | LINGUISTIC GROUP | COUNTRY | NUMBER SUBJECTS | SEX:  females - males | AGE^1^: mean (range) | % CITY^2^ | NUMBER SIBLINGS:  mean (range) | EDUCATION LEVEL^3^:  mean (range) | OCCUPATION^4^ | MONTHLY INCOME (€): mean (range) | MOST SPOKEN 2^ND^ LANGUAGE | KNOWLEDGE OPPOSITE BRANCHING^5^ |
| --- | --- | --- | --- | --- | --- | --- | --- | --- | --- | --- | --- | --- |
| Right | Italian | Italy | 30 | 11-19 | 37 (15-40) | 100 | 1  (0-2) | 14  (9-15) | 2-0-3-1-22-2 | 1780  (0-3500) | English | 0 |
|  | Khmer | Cambodia | 30 | 20-10 | 25 (15-43) | 0 | 3.8  (1-7) | 9  (3-12) | 11-3-6-2-0-8 | 41  (0-186) | English | 0 |
|  | Oshiwambo | Namibia | 30 | 20-10 | 25 (15-40) | 6.7 | 4.3  (0-11) | 7  (2-12) | 10-2-2-7-2-7 | 151  (0-874) | English | 0 |
|  | Northern Thai | Thailand | 30 | 16-14 | 28 (15-40) | 6.9 | 1.9  (0-6) | 12  (6-17) | 0-0-8-6-5-11 | 171  (0-389) | English | 0 |
|  |  |  |  | **67-53** | **29 (15-43)** | **28.6** | **2.7**  **(0-11)** | **10**  **(2-17)** | **23-5-19-**  **16-29-28** | **536**  **(0-3500)** | **English** | **0** |
| Left | Japanese | Japan | 30 | 16-14 | 21 (19-24) | 100 | 1.5  (0-3) | 15 | 0-0-0-0-0-30 | 0 | English | 1.6  (1-2) |
|  | Korean | South Korea | 24 | 14-10 | 22 (18-30) | 87.5 | 1.3  (0-2) | 15  (13-15) | 0-0-0-1-0-23 | 0 | English | 1.5  (1-2) |
|  | Nama | Namibia | 29 | 21-8 | 25 (14-40) | 13.8 | 4.9  (0-16) | 7  (0-15) | 7-2-3-5-0-12 | 156  (0-1036) | Afrikaans | 1.4  (1-2) |
|  | Sidaama | Ethiopia | 30 | 10-20 | 23 (16-35) | 76.7 | 7.2  (0-14) | 9  (0-15) | 5-2-6-3-0-14 | 18  (0-134) | Amharic | 0.2  (0-2) |
|  |  |  |  | **61-52** | **23 (14-40)** | **69.0** | **3.8**  **(0-16)** | **11**  **(0-15)** | **12-4-9-**  **9-0-79** | **45**  **(0-1036)** | **English** | **1.2**  **(0-2)** |

^1^ Age is in years

^2^ Percentage of people living in the city

^3^ Number of years of formal education

^4^ Number of participants being occupied in one of the following categories: unemployed participants; participants working in the primary sector; in the secondary sector; in the tertiary sector (commerce or tourism); in the tertiary sector (other services); students

^5^ Degree of knowledge of second languages with a branching opposite to the native language (from 0 to 2, according to a simplified version of the ILR scale)

**2. Site and language information**

- 1. Cambodia (Khmer)

Data were collected in a small village annexed to the town of Kratié, with approximately 40.000 inhabitants. Kratié is the capital of the Kratié province in northeastern Cambodia, and is located at the banks of the Mekong River. Kratié region has a monsoonal climate divided in a cool season from November to March, a hot season from March to May and a rainy season from May to October. The region is mostly covered by open deciduous tree forests interspersed with agricultural land, although the percentage of land devoted to agriculture is lower than elsewhere in Cambodia.

Most Kratié residents are either fishers or farmers, with the largest majority being employed in agriculture. Agriculture is mainly based on rice, cashew nut plantations and industrial crops like rubber. Most families live on a subsistence economy, and have little to no income. Transport is poorly developed in Kratié. It is connected by roads and ferries to the capital Phnom Penh and other large cities.

Ethnicity does not vary much in this region and the largest majority of inhabitants are Khmer, although the Kratié province is surrounded by indigenous ethnic minorities. The main religion is Theravada Buddhism and the first language is Khmer, as in the rest of the country. The town includes hospitals and 27 schools, including 2 schools that teach until grade 12. Although general education includes 6 years of primary school and 6 years of secondary general education, approximately 10% of children do not attend school at all and more than a half of Cambodian schoolchildren do not complete primary school at all. At school, classes are given in Khmer and pupils learn English as a second language.

According to Lewis and colleagues ^1^, Khmer is spoken by almost 15 million people as a first language, mostly in Cambodia. It is used in education, work, mass media and government at the national level. However, approximately one third of the population over 15 cannot read or write Khmer. Khmer belongs to the Austro-Asiatic family, to the Mon-Khmer subfamily and the Khmer genus. It is a strongly right-branching language, with a SVO order, head nouns preceding genitive and relative clauses, and separate adverbial subordinators at the beginning of subordinate clauses^24^.

- 1. Ethiopia (Sidaama)

Data were collected in Hawassa and at nearby outskirt rural compound. The small compound numbered few hundred people and was located 4km SW from the town of Hawassa. Hawassa is the regional capital of the Sidama zone, in the Southern Nations, Nationalities, and People´s Region (SNNPR), and is located at the eastern banks of the Awassa Lake, along the Rift Valley. The Sidama region has a mild climate, which is characteristic of the Ethiopian Highlands, with higher precipitations between July-September. The region is considered to be one of Ethiopia’s best agricultural areas, with most of the land oriented to that sector.

The Sidama population of Hawassa and the surrounding villages are mostly farmers, although a service and construction industry is developing in the urban area. Agriculture is largely based on false banana (enset), wheat and maize; the area also has a relevant number of livestock and produces 40% of the coffee in the country. However, most families live on a subsistence economy, having little to no income. Transport is poorly developed outside Hawassa and it depends on a precarious network of private collective vans. The city is connected by plane and a well paved road to the capital, Addis Ababa.

Around 50% of the inhabitants living in Hawassa are Sidama, although people with different ethnic and linguistic background are present in the city (e.g. Amhara, Welayta or Oromo). In the rural areas, even around the city, Sidama people represent above 90% of the total population. The main religion is protestant and the second one is Ethiopian Christian Orthodox, while the first language is Sidaama, which is the third language in Ethiopia in number of speakers (after Oromo and Amhara). The capital includes hospitals, several schools and universities.

General education includes 8 years of free and compulsory primary school and 4 years of secondary general education, which is not compulsory. An important percentage of the children abandon formal education after the primary period, which results in low fluency reading comprehension in Sidaama.

Sidaama is spoken by approximately 3 million people as a first language, mostly in Ethiopia ^1^. It is strongly used in its oral form and literature being widely sustained thanks to a system of institutionally supported education. Sidaama is taught in primary and secondary schools, used as a government working language and in radio programs. Sidaama belongs to the Afro-Asiatic family, to the Cushitic subfamily and to the Highland East Cushitic genus. It is a mainly left-branching language, with a SOV order and head nouns following genitive and relative clauses ^2^.

- 1. Italy (Italian)

Data were collected in Rome, the Italian capital. Rome is located in the central-western area of the Italian Peninsula, along the shores of Tiber river and close to the sea. Its history spans more than 2500 years and it is one of the oldest continuously occupied sites in Europe. To date, its metropolitan area has a population of 4.3 million residents. The city is characterized by a Mediterranean climate with cool humid winters and hot dry summers. Rome lays few meters above sea level and despite being a city also includes several green areas.

The economy of Rome largely depends on services, high-technology companies, research, construction, commercial activities, telecommunication and tourism, and it hosts the main national institutions and several international companies. Rome is served by an important network of roads and railways, three airports, a little harbor, and an extensive network of public transport.

Approximately 10% of the population consists of non-Italians, mainly from East Europe and Asia. Rome is largely Roman Catholic, and it has been an important religious center through the centuries. Natives speak standard Italian, although English is widely known in tourist areas and other languages are spoken in the abundant multilingual immigrant areas. The city includes more than 2000 schools and is an important center for higher education, with abundant colleges, academies and universities. Children have to attend school until they are 16, classes are given in Italian and pupils learn English (more rarely French or Spanish) as a second language.

According to Lewis and colleagues ^1^, Italian is spoken by more than 60 million people as a first language, mostly in Italy. It is used in education, work, mass media and government at the national level. The largest majority of Italians can use standard Italian and one of the numerous local dialects, switching from one to the other depending on what is most appropriate. Italian belongs to the Indo-European family and to the Romance genus. It is a strongly right-branching language, with a SVO order, head nouns preceding genitive and relative clauses, and separate adverbial subordinators at the beginning of subordinate clauses ^2^.

- 1. Japan (Japanese)

Data were collected in Kyoto, on the Honshu Island, in the region of Kanshai. With approximately 1.5 million inhabitants, Kyoto is one of the most important cities in Japan, and together with Kobe and Osaka it constitutes one of the largest built-up urban area in the world (with a total of 17 million inhabitants). The historical city is located in a valley, surrounded by mountains, and has a humid subtropical climate, with large differences in temperature across seasons, and between day and night.

The economy of Kyoto is mainly based on information technology, electronics and tourism (around 40% of the total employment corresponds to services; and around 37% of the total employment to industry ^3^). Kyoto is served by an extensive network of roads, railways, airports and public transport, making it a central hub in Japan and Asia.

In Kyoto, the vast majority of the population is Japanese, and the principal language spoken is Japanese. The predominant religions in this area are Buddhism and Shintoism. English is taught at school as a second language (together with Korean or German), although most people in Japan speak only rudimentary English. Kyoto is one of the main academic centers in Japan, and almost 10% of residents are students, with a growing number of international ones. Education is compulsory until the age of 15.

Japanese is spoken by more than 100 million people as a first language, mostly in Japan ^1^. It is used in education, work, mass media and government at the national level. Japanese belongs to the Japanese family and genus. It is a strongly left-branching language, with a SOV order, head nouns following genitive and relative clauses, and separate adverbial subordinators at the end of subordinate clauses ^2^.

- 1. South Korea (Korean)

Data were collected in two large cities, Gwangju and Seoul. Gwangju is a metropolitan city with approximately 1.5 million inhabitants, situated in the southwest of South Korea, close to the Gwangju River. The city is located between a mountainous area and a plains area. The city is characterized by a dry west coast climate, with high temperatures during summer and mild temperatures during winter.

Through history, the city has been a central place for commercial and cultural exchanges between people living in the mountains and plains areas. It is considered one of the best places in the country for agriculture, due to its soil and plains composition. Nowadays, Gwangju is a highly industrialized area with a great prominence of the automotive field, although its economy is still partly supported by the exportation of agricultural products.

Seoul is the capital and the largest city of South Korea, with more than 10 million inhabitants. It is located in the northwest of the country, in the basin of the Han River and surrounded by mountains. The city is usually classified as having a humid continental climate, with the monsoon season taking place during summer, and cold and dry climate during winter. Seoul went through an economic boom in the 60s, and today it is considered one of the most important hubs in the international economic system. Unemployment rate is low (3.8%) ^3^, despite being one of the most densely populated cities in Asia. Like Gwangju, Seoul is served by an airport and an extensive network of trains, buses and subway.

As most South Korean cities, Gwangju and Seoul are homogeneous societies with the vast majority of people being of Korean ethnicity. Among the non-Korean residents, most of them are Chinese. The main religions are Christianism and Buddhism, and half of the population is not religious. Usually individuals are introduced to foreign languages (mainly English or Chinese) during the elementary school years, around the age of 9. Both Gwanju and Seoul are important centers for higher education, with colleges, academies and universities. Children have to attend school until they are 15.

Korean is spoken by almost 80 million people as a first language, mostly in South Korea ^1^. It is used in education, work, mass media and government at the national level. Korean belongs to the Korean family and genus. It is a strongly left-branching language, with a SOV order, head nouns following genitive and relative clauses, and separate adverbial subordinators at the end of subordinate clauses ^2^.

- 1. Namibia (Nama)

Data were collected in Bethanie, a village in the !Karas region of southern Namibia. Bethanie is one of the oldest settlements in the country, with a population of about 2.000 individuals. The area around Bethanie originally belonged to the Red Nation and still plays an important role in the history and traditions of Nama people, hosting for instance the Nama Traditional Leaders’ Association, which groups the different Nama clans. Bethanie is surrounded by the !Karas mountain range in the South, the intermittent stream Konkiep on the West, and by mostly dry and rocky land. Daily temperatures are rather high, especially in summer, with little rainfall during the year.

Traditionally, Nama practiced a policy of communal land ownership, and the area around Bethanie is mainly characterized by small stock farms, with mostly goats and sheep. Unemployment rate is relatively high, but there are several mines and small scale industries in the region, where men go working. Tourist attractions are minor, and include a church and a little museum. Bethanie is relatively well connected by road to the larger center of Keetmanshoop, which has direct air, railway and road links with the Namibian capital of Windhoek.

Although there are several different ethnic groups in the area (e.g. Oshiwambo, Damara, San and Herero), the region is prevalently inhabited Nama. Schools are present in the village, and in the area around it. Education in Namibia is compulsory until the age of 16, but children in the poorest areas largely fail to attend school. In the last 20 years, English has been adopted as the main language of school instruction in most schools, but Nama remains by far the prevalent language spoken in the area, and older people have little to no knowledge of English, which is basically used only to interact with tourists. However, several people also use Afrikaans, whether in their households or as a lingua franca to interact with other ethnic groups.

Nama (also known as Khoekhoe) is spoken by more than 250.000 people as a first language, mostly in Namibia ^1^. It is a language recognized by the Ministry of Education since 2003 and it is strongly used in its oral form and literature being widely sustained thanks to a system of institutionally supported education. Nama is taught in primary and secondary schools, used in administration and radio programs. Nama belongs to the Khoe-Kwadi family and genus. It is a strongly left-branching language, with a SOV order, head nouns following genitive and relative clauses, and separate adverbial subordinators at the end of subordinate clauses ^2^.

- 1. Namibia (Oshiwambo)

Data were collected in the village of Olukonda, which was founded in 1870 by Finnish missionaries in the North of Namibia. It is the district capital of the Olukonda electoral constituency, which counts less than 10.000 inhabitants. Although it is part of Namibia, Olukonda also belongs to the traditional kingdom of Ondonga. The area is characterized by a hot semi-arid climate, with little rainfall during the year.

The area around Olukonda is mainly agricultural, although cattle rearing is also common and unemployment is very high. Olukonda has a church and a mission station with a museum and a rest camp, which are a community-based tourism institution providing work to several people in the village. Communication is relatively good in much of the area, with a paved trunk road linking the village to the north and the south of the country, but basically no public transport.

Although there are several different ethnic groups in the area (including Damara, Nama, San and Herero), the region is prevalently inhabited by the Oshiwambo. Traditionally, the Oshiwambo had a complex system of religious beliefs and magical practices, but as a result of missionaries most traditional beliefs have died out, and people have integrated traditional beliefs into a syncretic Christianity. The village of Olukonda has more than five schools. Although education in Namibia is compulsory until the age of 16, not all children attend school, especially in the poorest areas. In most schools, English has been adopted as the main language of school instruction in the last 20 years. However, Oshiwambo remains by far the prevalent language spoken in the area, and older people have little to no knowledge of English, which is basically used only to interact with tourists and other ethnic groups.

Oshiwambo (also known as Ndonga) is spoken by more than 800.000 people as a first language, mostly in Namibia ^1^. It is a language recognized by the Ministry of Education since 2003, which is strongly used in its oral form, but only limitedly in literature. Oshiwambo belongs to the Niger-Congo family, to the Benue-Congo subfamily and to the Bantoid genus. It is a strongly right-branching language, with a SVO order, head nouns preceding genitive and relative clauses, and separate adverbial subordinators at the beginning of subordinate clauses ^2^.

- 1. Thailand (Northern Thai)

Data were collected in Chiang Mai, which is the main city in Northern Thailand. Its foundation dates back to the 13^th^ century. Chiang Mai is located along the Ping River. To date, its metropolitan area has a population of nearly one million residents, extending into numerous neighbouring districts. The city is characterized by a tropical wet and dry climate, tempered by the elevation and low latitude, with warm to hot weather throughout the year.

The economy of Chiang Mai significantly changed in the last years. Although it mainly relied on a heavy-industry-based economic, the city is now turning toward tertiary, also developing as a major touristic city in South East Asia. Unemployment rate is relatively high and several people live below the poverty line. Chiang Mai is served by an important network of roads, a navigable river, an international airport, railways and bus service, as well as an extensive network of local public transport.

The main ethnic group in Chiang Mai is Thai, although numerous minorities live in the surrounding hills and partially in the city, including the Karen, Lahu, Hmong, Lisu, Akha, Mien, and Padaung. The main religion is Theravada Buddhism, with Chiang Mai having over 300 Buddhist temples. Natives speak Northern Thai, although central Thai is used in education. English is widely known in tourist areas and other languages are spoken in the immigrant areas. The city includes several museums and cultural centers, several universities and colleges. Education is free and compulsory until 15 years, and English is taught at school as a second language.

Northern Thai is spoken by more than 6 million people as a first language, the largest majority living in Northern Thailand ^1^. Most people use Northern Thai at home, which is strongly used in its oral form, but only limitedly in literature. Northern Thai belongs to the Tai-Kadai family and to the Kam-Tai genus. It is a strongly right-branching language, with a SVO order, head nouns preceding genitive and relative clauses, and separate adverbial subordinators at the beginning of subordinate clauses ^2^.

**3. Experimental protocol**

Testing took place in surroundings that were familiar to the participants, such as schools, community centers and private homes. Individuals were generally tested alone, unless they felt uncomfortable and asked for other people being present, in which case they were sat at a certain distance and instructed not to interfere in any way with the testing procedure. For each population, one research assistant collected the data together with a local research assistant translating the procedure, when needed (i.e. in Cambodia, Ethiopia, Japan, Korea and Namibia). In this way, recruiting, consenting and testing were always conducted in the local language. Participants were recruited in community centers and through community contacts. Written consent was obtained before testing.

Each participant was tested in 6 different memory tasks, administered one after the other on a laptop, with little breaks in-between. The order in which tasks were presented was pseudo-randomized across subjects in all languages. The six tasks were three short-term memory (STM) tasks with words as stimuli (WS=word span), with numbers as stimuli (DS=digit span), or with spatial stimuli (MS=matrix span); and three working memory (WM) tasks with words as stimuli (OS=operation span), with numbers as stimuli (CS=counting span), or with spatial stimuli (SS=symmetry span) (Figure S1). For these tasks, we adapted the automated span tasks programmed with E-prime and implemented by Unsworth and colleagues ^4-5^. All tasks basically require individuals to observe a series of stimuli and recall them immediately afterwards, in the same order they were presented. Before each task started, participants were instructed about the procedure and provided with two examples containing two stimuli (see below). In case the procedure was not clear, it was explained again until the participant understood it. Throughout the tasks, the experimenter made no suggestions, but could motivate participants regardless of their performance by reassuring them that they were doing fine.


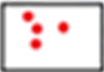

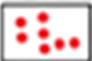


**
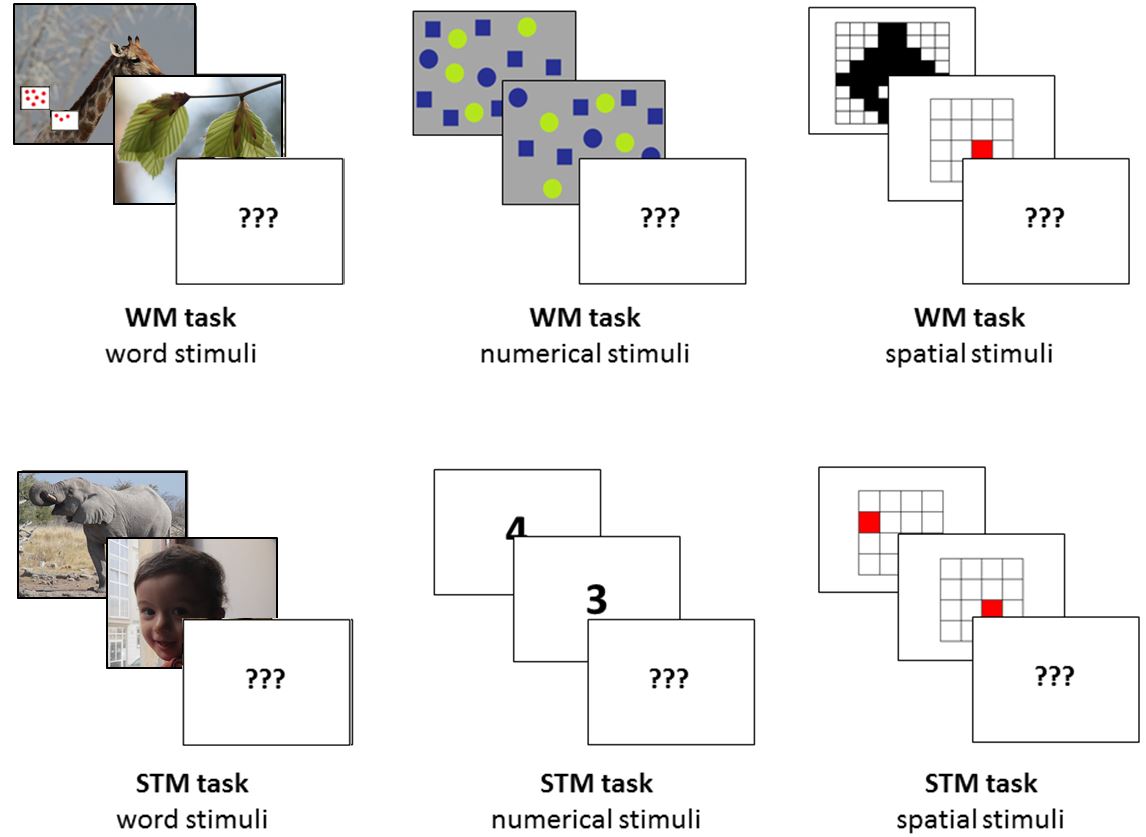
**

**Figure S1**. Pictorial representation of the six tasks administered.

- 1. STM-WS task: short-term memory with word stimuli

In the STM-WS task, participants were presented with 18 test trials. Trials contained 2, 3, 4, 5, 6 or 7 stimuli. We presented three trials for each number of stimuli, pseudo-randomizing the order of trials. The stimuli used in each trial were the same for all individuals and languages (with the exceptions listed at the end of this section), and none was used more than twice in the task. The stimuli consisted of 600 _px_ x 800 _px_ pictures, with images of common animals and objects (e.g. a cat, a hen, a leaf, an ant, a cloth).

Before the task started, individuals were instructed to observe the series of pictures on the screen, name each of them aloud as soon as it appeared, and recall them loud in the same order they had appeared, as soon as question marks appeared on the screen. The experimenter especially emphasized the importance of recalling the stimuli in order, providing examples. Moreover, participants were explained that stimuli could be named in the way participants considered to be more correct (e.g. the image of an ant could be called “ant”, but also “insect”), provided they used the same name when recalling them at the end of the trial. Traditionally, this task presents subjects with written words that need to be read aloud and later recalled. However, it is common in cross-linguistic and cross-cultural research to use images instead of written words in order to account for the different degree of literacy across ethnic groups and also allow participation to people with little to no education ^6-7^.

At the beginning of each trial, the experimenter drove the participant’s attention to the laptop and then made the stimuli start. Participants saw several stimuli (each one being visible for 2 seconds in the middle of the screen) and named them as they saw them on the screen. When the question marks appeared, they recalled the stimuli aloud. If participants changed their answer right after recalling the stimuli and before the following trial started, the last answer was coded. The experimenter audio-recorded all trials.

We later coded trials from the audio files, transcribing for each trial the name of the stimuli recalled in the order they were recalled. We then compared the recalled stimuli to the stimuli as named during the stimuli presentation (if some stimuli were not named during the stimuli presentation, we compared the recalled stimuli to the name most frequently given to the stimulus by the other participants of the same linguistic group). For each trial, we divided the list of stimuli presented in two halves (if the number of stimuli presented was uneven, we ignored the middle one), and separately coded the number of correct responses for the first half (i.e. initial stimuli) and the second half (i.e. final stimuli). For the first half, we coded whether the first stimulus recalled corresponded to the first stimulus having been presented, whether the second stimulus recalled corresponded to the second stimulus having been presented, and so on. For instance, if subjects were presented with the stimuli ABCDE in a trial, and recalled ACBE, they would have scored 1 for the initial stimuli, as the first recalled stimulus A corresponded (in identity and position) to the first stimulus A having been presented, but C did not correspond to B. For the second half, we coded whether the last stimulus recalled corresponded to the last stimulus having been presented, the second to last stimulus recalled corresponded to the second to last stimulus having been presented, and so. For instance, if subjects were presented with the stimuli ABCDE in a trial, and recalled ACBE, they would have scored 1 for the final stimuli, as the last recalled stimulus E corresponded (in identity and position) to the last stimulus E having been presented, but B did not correspond to D. Crucially, coding the final stimuli starting from the end ensured that mistakes in recalling initial stimuli did not affect the response for the final stimuli, as a correct response required that both identity and order of stimuli were recalled correctly: forgetting an initial stimulus but recalling all the final ones correctly would have resulted in a wrong assessment of performance for the final stimuli, had we coded them from the beginning like for the initial stimuli. For instance, if subjects were presented with the stimuli ABCDE in a trial, and recalled ACBE, they would have scored 0 for the final stimuli, had we traditionally coded from the beginning, as the third recalled stimulus B did not correspond to the third stimulus C having been presented, and the fourth recalled stimulus E did not correspond to the fourth stimulus D having been presented, resulting in a 0 score for the final stimuli, although E had been correctly recalled.

- 1. STM-DS task: short-term memory with numerical stimuli

In the STM-DS task, participants were presented with 21 test trials. Trials contained 3, 4, 5, 6, 7, 8 or 9 stimuli. We presented three trials for each number of stimuli, pseudo-randomizing the order of trials. The stimuli used in each trial were the same for all individuals and languages, and each of them was used 13 or 14 times in the task. The stimuli consisted of numbers (from 1 to 9, as 100 _px_ x 150 _px_ images with a black number on a white background).

Before the task started, individuals were instructed to observe the series of numbers on the screen and recall them in the same order they had appeared, as soon as question marks appeared on the screen, by writing them down in a coding sheet already provided by the experimenter. The experimenter especially emphasized the importance of recalling the stimuli in order, providing examples. The coding sheets consisted of a series of 9 squares close to each other, so that a number could be written in each square. Being always 9 squares, participants were not provided with any information as to the number of stimuli to be recalled in that specific trial, as in the other tasks. For each trial, a different coding sheet was provided, so that numbers written in the previous trial did not visually interfere with those recalled in the following trial.

At the beginning of each trial, the experimenter drove the participant’s attention to the laptop and then made the stimuli start. Participants saw several stimuli (each one being visible for 2 seconds in the middle of the screen) and when the question marks appeared, they wrote down the recalled the stimuli on the coding sheet. If participants changed their answer right after recalling the stimuli and before the following trial started, by noting it down on the coding sheet, the last answer was coded.

We later coded trials from the coding sheets, comparing the stimuli written in the coding sheet with the stimuli they had been presented in each trial. For each trial, we divided the list of stimuli presented in two halves (as explained above), and separately coded the number of correct responses for the first half (i.e. initial stimuli) and the second half (i.e. final stimuli) of the stimuli, to see if the stimuli recalled corresponded to the stimuli having been presented.

- 1. STM-MS task: short-term memory with spatial stimuli

In the STM-MS task, participants were presented with 18 test trials. Trials contained 2, 3, 4, 5, 6 or 7 stimuli. We presented three trials for each number of stimuli, pseudo-randomizing the order of trials. The stimuli used in each trial were the same for all individuals and languages, and each of them was used no more than 5 times in the task. The stimuli consisted of 4 x 4 squared matrixes with a black grid on a white background, and one of the 16 squares inside being colored red in each stimulus (the position of this red square was different depending on the stimulus). Matrixes were presented as 400 _px_ x 300 _px_ images.

Before the task started, individuals were instructed to observe the series of matrixes on the screen and recall the position of each red square in the same order they had appeared, as soon as question marks appeared on the screen, by writing them down in a coding sheet already provided by the experimenter. The experimenter especially emphasized the importance of recalling the stimuli in order, providing examples. The coding sheets consisted of a series of seven 4 x 4 matrixes similar to the ones observed on the screen, so that the position of the red square in each matrix observed could be marked with a cross or sign on each matrix of the coding sheet. Being always seven matrixes, participants were not provided with any information as to the number of stimuli to be recalled in that specific trial, as in the other tasks. For each trial, a different coding sheet was provided, so that matrixes in the previous trial did not visually interfere with those recalled in the following trial.

At the beginning of each trial, the experimenter drove the participant’s attention to the laptop and then made the stimuli start. Participants saw several stimuli (each one being visible for 2 seconds in the middle of the screen) and when the question marks appeared, they wrote down the recalled the stimuli on the coding sheet. If participants changed their answer right after recalling the stimuli and before the following trial started, by noting it down on the coding sheet, the last answer was coded.

We later coded trials from the coding sheets, comparing the stimuli written in the coding sheet with the stimuli they had been presented in each trial. For each trial, we divided the list of stimuli presented in two halves (see above), and separately coded the number of correct responses for the first half (i.e. initial stimuli) and the second half (i.e. final stimuli) of the stimuli, to see if the stimuli recalled corresponded to the stimuli having been presented.

- 1. WM-OS task: working memory with word stimuli

In the WM-OS task, participants were presented with 12 test trials. Trials contained 2, 3, 4 or 5 stimuli. We presented three trials for each number of stimuli, pseudo-randomizing the order of trials. The stimuli used in each trial were the same for all individuals and languages (with the exceptions listed at the end of this section), and each was used only once in the task. The stimuli consisted of 600 _px_ x 800 _px_ pictures with images of common animals and objects (as in the STM-WS task), and three little squares with a variable number of red dots inside, on the bottom of the picture (so not to cover the image), which instead served as stimuli for the distracting task.

Before the task started, individuals were instructed to observe the series of pictures on the screen, name each of them aloud as soon as it appeared, solve the distracting task (by subtracting the red dots in a box from the red dots in the other one, and telling aloud whether the result corresponded to the number of red dots in the third box; i.e. distracting task), and recall the name of the pictures loud in the same order they had appeared, as soon as question marks appeared on the screen. The experimenter especially emphasized the importance of recalling the word stimuli in order, providing examples. Moreover, participants were explained that word stimuli could be named in the way participants considered to be more correct (see above), provided they used the same name when recalling them at the end of the trial. Traditionally, this task presents subjects with written words to be recalled, and a distracting task with mathematically written operations, but we refrained from using written words and mathematically written operations to take into account the different degree of literacy across ethnic groups and also allow participation to people with little to no education (see above).

At the beginning of each trial, the experimenter drove the participant’s attention to the laptop and then made the stimuli start. Participants saw several stimuli (each of them remaining in the middle of the screen until it was named and the mathematical operation was solved) and named them as they saw them on the screen, while also solving the mathematical operation. When the question marks appeared, they recalled the word stimuli aloud. If participants changed their answer right after recalling the word stimuli and before the following trial started, the last answer was coded. The experimenter audio-recorded all trials. We later coded trials from the audio files, transcribing for each trial the name of the word stimuli recalled in the order they were recalled, and the response to the distracting task. Later, we coded whether the response to the distracting task was correct, and entered the percentage of correct choices in each trial of the distracting task as a fixed effect in the statistical model (see below). We further compared the recalled stimuli to the stimuli as named during the stimuli presentation. For each trial, we divided the list of word stimuli presented in two halves (see above), and separately coded the number of correct responses for the first half (i.e. initial stimuli) and the second half (i.e. final stimuli) of the word stimuli, to see if the stimuli recalled corresponded to the stimuli having been presented.

- 1. WM-CS task: working memory with numerical stimuli

In the WM-CS task, participants were presented with 15 test trials. Trials contained 2, 3, 4, 5 or 6 stimuli. We presented three trials for each number of stimuli, pseudo-randomizing the order of trials. The stimuli used in each trial were the same for all individuals and languages, and each of them was used only once in the task. The stimuli consisted of 600 x 800 pictures with a grey background and a varying number of blue circles, blue squares and green circles (with the number of blue circles in each image varying from 3 to 9).

Before the task started, individuals were instructed to observe the series of images on the screen, count aloud the number of blue circles among other figures in each image (i.e. distracting task), repeat it loud and recall aloud the series of final numbers in the same order they had appeared (as in the STM-DS task), as soon as question marks appeared on the screen. The experimenter especially emphasized the importance of recalling the numeric stimuli in order, providing examples.

At the beginning of each trial, the experimenter drove the participant’s attention to the laptop and then made the stimuli start. Participants saw several stimuli (each of them remaining in the middle of the screen until the blue circles had been counted) and for each stimulus they repeated the number of blue circles aloud. When the question marks appeared, they recalled the numeric stimuli (i.e. the number of blue circles) aloud. If participants changed their answer right after recalling the numeric stimuli and before the following trial started, the last answer was coded. The experimenter audio-recorded all trials. We later coded trials from the audio files, transcribing for each trial the numbers recalled in the order they were recalled, and the response to the distracting task (i.e. the number of blue circles having been counted in each trial, when the image was still present). Later, we coded whether the response to the distracting task was correct, and entered the percentage of correct choices in each trial of the distracting task as a fixed effect in the statistical model (see below). We further compared the recalled numeric stimuli to the stimuli as named during the stimuli presentation (i.e. to the number of blue circles having been counted in each trial). For each trial, we divided the list of stimuli presented in two halves (see above), and separately coded the number of correct responses for the first half (i.e. initial stimuli) and the second half (i.e. final stimuli) of the numeric stimuli, to see if the stimuli recalled corresponded to the stimuli having been presented.

- 1. WM-SS task: working memory with spatial stimuli

In the WM-SS task, participants were presented with 12 test trials. Trials contained 2, 3, 4 or 5 stimuli. We presented three trials for each number of stimuli, pseudo-randomizing the order of trials. The stimuli used in each trial were the same for all individuals and languages, and each of them was used 2 to 3 times in the task. The stimuli consisted of 4 x 4 squared matrixes with a black grid on a white background (as in the STM-MS task), and one of the 16 squares inside being colored red in each stimulus (the position of this red square was different depending on the stimulus). Four x four matrixes were presented as 400 _px_ x 300 _px_ images, and were alternated to 8 x 8 squared matrixes of the same size, which instead serves as stimuli for the distracting task. In 8 x 8 matrixes some of the 64 squares were colored black, forming a muster that could either be symmetrical or asymmetrical along the vertical axis (with 8 x 8 matrixes differing in all stimuli and trials, half being symmetrical and half not).

Before the task started, individuals were instructed to observe the series of 4 x 4 matrixes on the screen, assess aloud whether the 8 x 8 symmetry matrixes were symmetrical or not (i.e. distracting task), and recall the position of each red square in the 4 x 4 matrixes in the same order they had appeared, as soon as question marks appeared on the screen, by writing them down in a coding sheet already provided by the experimenter. The experimenter especially emphasized the importance of recalling the spatial stimuli in order, providing examples. The coding sheets consisted of a series of seven 4 x 4 matrixes similar to the ones observed on the screen, so that the position of the red square in each 4 x 4 matrix observed could be marked with a cross or sign on each matrix of the coding sheet. Being always five matrixes, participants were not provided with any information as to the number of stimuli to be recalled in that specific trial, as in the other tasks. For each trial, a different coding sheet was provided, so that matrixes in the previous trial did not visually interfere with those recalled in the following trial.

At the beginning of each trial, the experimenter drove the participant’s attention to the laptop and then made the stimuli start. Participants saw several stimuli (each 4 x 4 matrix being visible for 2 seconds in the middle of the screen, followed by a 8 x 8 symmetry matrix also being visible for 2 seconds each in the middle of the screen), and when the question marks appeared, they wrote down the recalled spatial stimuli on the coding sheet. If participants changed their answer right after recalling the spatial stimuli and before the following trial started, by noting it down on the coding sheet, the last answer was coded. On a piece of paper, the experimenter noted down the responses to the distracting task (i.e. whether the 8 x 8 symmetry matrix was assessed to be symmetrical or not). Later, we coded whether the response to the distracting task was correct, and entered the percentage of correct choices in each trial of the distracting task as a fixed effect in the statistical model (see below). We further compared the 4 x 4 matrixes entered in the coding sheet as a response, with the spatial stimuli they had been presented in each trial. For each trial, we divided the list of stimuli presented in two halves (see above), and separately coded the number of correct responses for the first half (i.e. initial stimuli) and the second half (i.e. final stimuli) of the spatial stimuli, to see if the stimuli recalled corresponded to the stimuli having been presented.

- 1. Questionnaire

At the end of all the tasks, participants were asked their name, sex and age, whether they lived in the village or in the city, the number of siblings they had, their main occupation and approximate monthly income, their educational level, their native language, whether they spoke other languages (and if so, which ones, when they started learning them, how/where they learned them, how often and where/with whom they used them, and what they could do with them; see below).

- 1. Modification of stimuli between sites

To ensure maximal comparability, the same stimuli were used for all participants and linguistic groups. However, the following exceptions were made to ensure that stimuli also had a similar valence across groups:

1. Ethnicity of baby, child and family was always matched to the participants (i.e. White Caucasian people in Italy, vs. East-Asian people in Cambodia, Japan, South Korea and Thailand, etc.);
2. Coffee was used in Italy, vs. beans in Cambodia, Japan, Namibia, South Korea and Thailand;
3. Lion was used in Italy, vs. jaguar in Namibia, vs. tiger in Cambodia, Japan, South Korea and Thailand;
4. Horse was used in Cambodia, Italy, Japan, South Korea and Thailand, vs zebra in Namibia;
5. Hyena was used in Italy, vs. jackal in Cambodia, Japan, Namibia, South Korea and Thailand;
6. Ant was used in Cambodia, Italy, Japan, South Korea and Thailand, vs. termite in Namibia;
7. Hen was used in Cambodia, Italy, Japan, South Korea and Thailand, vs. ostrich in Namibia.

**4. Inter-observer reliability**

To assess the accuracy of the experimenter’s coding, a second observer re-coded 11.6% of all the trials. The trials to re-code were randomly selected and were similarly distributed for all the 6 tasks and linguistic groups. Inter-observer reliability was excellent (for the sum of correct initial stimuli in each trial: Cohen’s k = .955, N = 2592, *p* < .001; for the sum of correct final stimuli in each trial: Cohen’s k = .940, N = 2592, *p* < .001).

**5. Model analyses**

Before conducting the analyses, we excluded some trials. In particular, although all participants alleged to be native speakers of a single language, based on the interactions with the participants we inferred that one Korean and one Nama-speaker were not native speakers of those languages and we therefore dropped them from the analyses. We further excluded from the analyses one Sidaama who failed to count the blue circles aloud in the distracting task of the WM-CS task (as the distracting task was therefore not implemented, transforming the nature of the WM task). Finally, we excluded 68 trials (i.e. 0.3% of the remaining trials), due to problems with the audio-recordings, participant’s failure to understand the procedure, participant’s distraction or others’ interference in the task.

All analyses were conducted using generalized linear mixed models (GLMM ^8^) and were run using R statistics (version 3.2.3) with the lme4 package ^9^. Models were run with a Poisson structure. All numerical variables were z-transformed. To analyze whether multiple test predictors influenced a response, full models were compared to a null model excluding the test variables. Before assessing the effect of individual predictors, we inspected the significance of the full model as a whole. This is an important practice, because the probability that one predictor reveals significant increases with the number of predictors in the model, increasing the likelihood of Type I errors ^10^. The full model included all test predictors of interest, all the control predictors and all possible interactions. The null model lacked the predictors of interest. If the full-null model comparison reveals significant, it means that the full model explains the response significantly better than the null model, i.e. one or more predictors of interest have a significant impact on the response. To obtain the *p* values for the individual fixed-effects we conducted likelihood-ratio tests ^11^.

For every model we assessed model stability by comparing the estimates derived by a model based on all data with those obtained from models with the levels of the random effects excluded one at a time. All models were stable. In order to rule out collinearity, we checked variance inflation factors (VIF ^12^), and overall VIF values were close to one (maximum VIF = 3.26).

5.1 Test predictors for STM and WM models

The following model predictors were included in the models:

- branching direction (2 levels: right or left);
- kind of stimuli (3 levels: numerical, spatial and word);
- stimuli position within each trial (2 levels: initial or final).

“Branching direction” was determined depending on the native language of the participants and according to literature ^2^. “Kind of stimuli” referred to the stimuli used in each task, which could either be numbers, spatial matrixes or words. “Stimuli position” referred to the position of the stimuli within a trial, as for each trial stimuli were divided into initial and final ones (i.e. the first and the second half in the list of stimuli, excluding the central stimulus for trials with an uneven number of stimuli).

5.2 Control predictors: fixed effect variables for all models

The following fixed effect variables (i.e. covering the entire population of the levels of a factor) were included in the models:

- participant’s sex (2 levels);
- participant’s age (range from 14 to 43 years old);
- number of siblings (from 0 to 16);
- residence (2 levels: village/town, i.e. <100.000 inhabitants, or city, i.e. >100.000); as the people we tested were not necessarily residing in the place they were tested (so that in the villages we also tested participants residing in a city, or vice versa), this predictor was not nested within language;
- education (18 levels: depending on the number of years spent at school/University);
- occupation (6 levels: unemployed participants (0), participants working in the primary sector (1), participants working in the secondary sector (2), participants working in the tertiary sector in commerce or tourism (3), participants working in the tertiary sector in other services (4), and students (5));
- centered income (calculated for each participant as the deviation of each participant’s monthly income from the average national income);
- average national income (average national monthly income in each country, from 58 to 2588 €);
- knowledge of a language with an opposite branching (3 levels: no knowledge (0), low to middle knowledge (1), middle to high knowledge (2));
- number of stimuli in each trial (from 2 to 9, depending on the trial and task);
- trial number within each task (from 1 to 21, depending on the task).

Average national income was taken on the 16.11.2015 from the ILO website: http://www.ilo.org/ilostat/faces/home/statisticaldata/ContryProfileId?_adf.ctrl-state=j3jo7udvn_636&_afrLoop=269696306794527, and then transformed from local currencies into euros on the same day. Knowledge of a language with an opposite branching was assessed in the following way. Firstly, during the questionnaire we collected all the information about any other language that participants could speak. In order to do that, we used a modified version of the ILF (Interagency Language Roundtable) scale for Language Proficiency (http://www.govtilr.org), the standard language proficiency test by the U.S. Department of State. The scale for Language Proficiency contains 6 levels (0 to 5). We used the first three levels of the this scale (0 to 2) so that our levels 0 and 1 corresponded to the original 0 and 1 levels, and our level 2 grouped all levels equal or higher than 2. Possible non-native speakers were excluded from the final sample (see above). Knowledge of the second language was assessed by each of the experimenters that collected the data, with the help of a local translator when necessary. Secondly, for each second language we assessed its main branching direction based on (i) the SVO/SOV order, the presence of head nouns preceding/following (ii) genitive and (iii) relative clauses, and (iv) separate adverbial subordinators at the beginning/end of subordinate clauses^2^. In the model, we included the degree of knowledge of all the second languages having a branching direction mainly going in the opposite direction of the native language (as this could have interfered with the psycholinguistic effects of the native language).

Furthermore, the WM model also contained the percentage of correct choices in the distracting trials as a control variable (fixed effect). In the STM model, for the STM-WS task we also aimed to include as fixed effect whether participants named at least one stimulus aloud when watching the images in a trial. However, this was not possible, because such a fixed effect was only present in the STM-WS task, and thus only affected one predictor level (i.e. kind of stimuli (word)).

5.3 Control predictors: random effect variables for all models

The inclusion of random effects (i.e. those representing only a randomly chosen sample out of the possible levels of a factor) is necessary to control for repeated observations. Repeated observations of an individual or a language, otherwise, can lead to non-independent clusters of residuals, violating the assumption of independent residuals. The following random effects were included in the model:

- Language;
- Participant’s identity;
- Trial identity: instead of modeling the difference in performance directly, the dependent variable for the model was the number of correct stimuli for a specified position within a trial (i.e. from initial or final position). This resulted in 2 data points per trial, one for the initial position and one for the final position. Position (initial or final) was included in the model, such that a significant effect of position would suggest a change in performance from beginning to end. To account for the non-independence of each pair of data points, trial identity was therefore included in the model as a random effect.

5.4 Model 1: the WM model

Model 1 investigated whether participants correctly recalled the stimuli in the WM tasks. In this model we included participants’ performance in WM numerical, spatial and word tasks (N = 18050). The dependent variable was the number of correct stimuli identified. Thus, the full model included branching direction, kind of stimuli, and stimuli position as test predictors, as well as the interaction between branching, kind of stimuli and stimuli position (3-way-interaction). We further included the control predictors sex, age, number of siblings, residence, education, occupation, centered income, average national income, knowledge of a language with an opposite branching, number of stimuli in each trial, trial number within each task and the percentage of correct choices in the distracting trials as fixed effects, including all the possible random slopes (also for the test predictors). We further included participant’s identity, language, and trial identity as random effects. The comparison between the full model and the null model was significant (GLMM: *p* < 0.001, N = 18050, *χ*^2^_11_ = 55.81). We dropped the non-significant three-way interaction from the model (branching*kind of stimuli*position of stimuli) (GLMM: *p* = 0.65, N = 18050, *χ*^2^_2_ = 0.86) and the non-significant two-way interaction branching*kind of stimuli (GLMM: *p* = 0.41, N = 18050, *χ*^2^_2_ = 1.79). We found two significant two-way interactions (Figures 1 and Figure S2). A first two-way interaction between kind of stimuli and position of stimuli revealed that participants were better at recalling number and word stimuli in the final position, but worse at recalling spatial stimuli in the final position (GLMM: *p* < 0.001, N = 18050, *χ*^2^_2_ = 23.45). We also found a second two-way interaction between branching and position of stimuli. In particular, left branching participants were better than right branching participants at recalling initial stimuli, but were worse at recalling final stimuli (GLMM: *p* = 0.01, N = 18050,*χ*^2^_1_ = 6.48) (see Table S2).


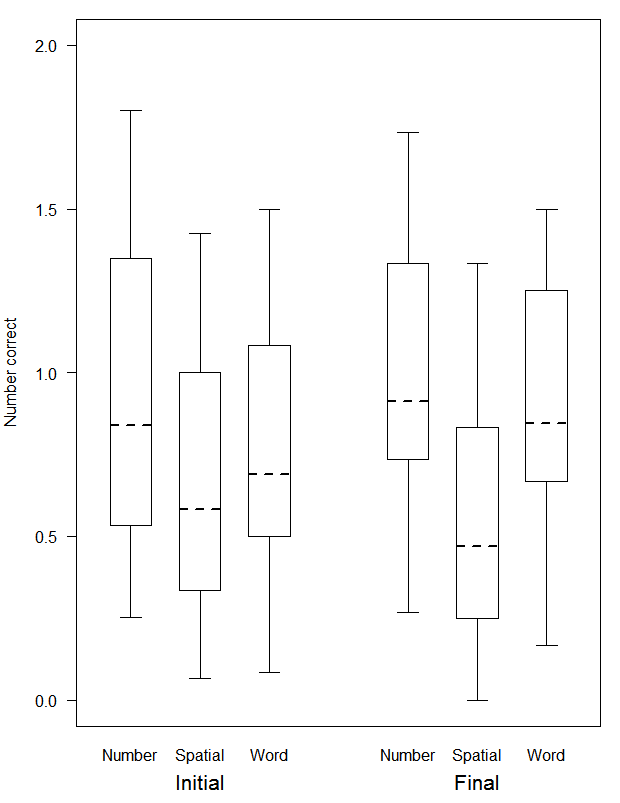


**Figure S2**. Box-plot representing the data distribution for the number of correct initial and final stimuli across the three kinds of stimuli from Model 1 (GLMM). The horizontal ends of the box represent the 75% and 25% quartiles, and the ends of the whiskers represent the 97.5% and 2.5% quartiles respectively. The dotted line represents the model estimates.

5.5 Model 2: the STM model

Model 2 investigated whether participants correctly recalled stimuli in the STM tasks. In this model we included participants’ performance in STM numerical, spatial and word tasks (N = 26470). The dependent variable was the number of correct stimuli identified. Thus, the full model included branching direction, kind of stimuli and stimuli position as test predictors, as well as the interaction between branching, kind of stimuli and stimuli position (3-way-interaction). We further included the control predictors sex, age, number of siblings, residence (city or village), level of education, occupation, centered income, average national income, knowledge of a language with an opposite branching, number of stimuli in each trial, and trial number within each task as fixed effects, including all the possible random slopes (also for the test predictors). We further included participant’s identity, language and trial identity as random effects. The comparison between the full model and the null model was significant (GLMM: *p* < 0.001, N = 26470, *χ*^2^_11_ = 78.79). We dropped the non-significant three-way interaction (branching*kind of stimuli*position of stimuli) (GLMM: *p* = 0.19, N = 26470, *χ*^2^_2_ = 3.23) from the model. We also dropped the two non-significant two-way interactions from the model: branching*kind of stimuli (GLMM: *p* = 0.42, N = 26470, *χ*^2^_2_ = 1.71) and branching*stimuli position (GLMM: *p* = 0.78, N = 26470, *χ*^2^_1_ = 0.076). We found a significant two-way interaction between kind of stimuli and position of stimuli (Figure S3). In particular, participants were better at recalling initial stimuli as compared to final stimuli in all tasks and this effect was steeper in number stimuli compared to spatial and word stimuli (GLMM: *p* < 0.001, N = 26470, *χ*^2^_2_ = 27.42) (see Table S3).

**
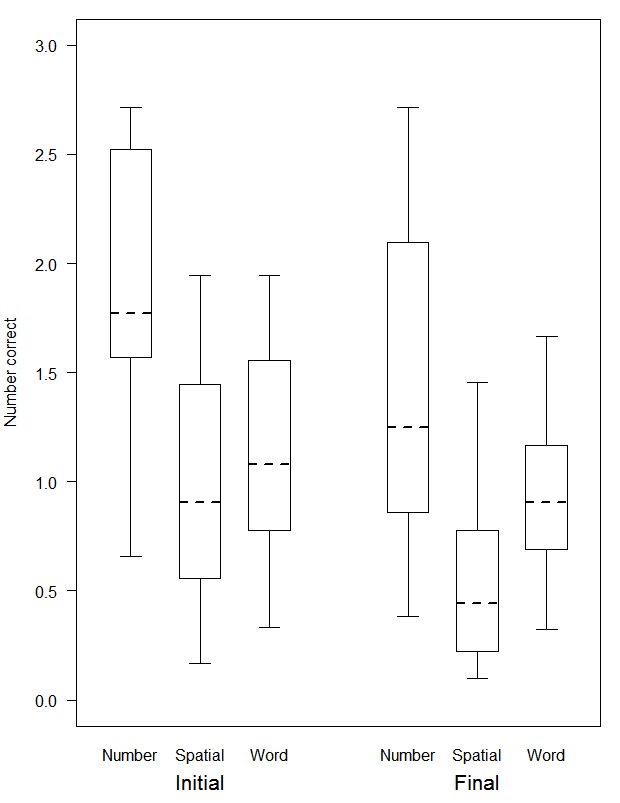
**

**Figure S3**. Box-plot representing the data distribution for the number of correct initial and final stimuli across the three kinds of stimuli from Model 2 (GLMM). The horizontal ends of the box represent the 75% and 25% quartiles, and the ends of the whiskers represent 97.5% and 2.5% quartiles respectively). The dotted line represents the model estimates.

**Table S2**. Results for the WM Model. In bold, significant results. For details on the test categories, please see the text. In case interactions were tested, the main effects were also included in the model, but are not listed below.

| **Test Category** | **Estimate** | **SE** | **Chi-square** | **df** | ***P*** | **CI (95%) of the reduced model** |
| --- | --- | --- | --- | --- | --- | --- |
| Intercept | -0.114 | 0.145 | - | - | - | -0.558 / 0.283 |
| Sex | -0.029 | 0.045 | 0.39 | 1 | 0.53 | -0.158 / 0.098 |
| Age | -0.023 | 0.026 | 0.68 | 1 | 0.41 | -0.095 / 0.454 |
| Number of siblings | 0.015 | 0.022 | 0.49 | 1 | 0.48 | -0.045 / 0.074 |
| Residence | -0.023 | 0.059 | 0.1 | 1 | 0.75 | -0.204/ 0.167 |
| **Education** | **0.076** | **0.027** | **6.48** | **1** | **0.01** | **-0.001 / 0.155** |
| Occupation 1 | 0.098 | 0.127 | 2.33 | 5 | 0.8 | -0.275 / 0.438 |
| Occupation 2 | 0.128 | 0.1 | 2.33 | 5 | 0.8 | -0.155 / 0.408 |
| Occupation 3 | 0.076 | 0.099 | 2.33 | 5 | 0.8 | -0.2 / 0.358 |
| Occupation 4 | -0.065 | 0.201 | 2.33 | 5 | 0.8 | -0.642 / 0.533 |
| Occupation 5 | 0.044 | 0.082 | 2.33 | 5 | 0.8 | -0.198 / 0.276 |
| Centered income | -0.055 | 0.055 | 0.78 | 1 | 0.38 | -0.214 / 0.101 |
| **Average national income** | **0.186** | **0.081** | **4.08** | **1** | **0.04** | **0.037 / 0.4** |
| Knowledge of another language 1 | -0.053 | 0.102 | 0.99 | 2 | 0.61 | -0.364 / 0.244 |
| Knowledge of another language (2) | -0.093 | 0.109 | 0.99 | 2 | 0.61 | -0.416 / 0.224 |
| Number of stimuli | 0.053 | 0.036 | 1.84 | 1 | 0.17 | -0.051 / 0.149 |
| Trial number | -0.003 | 0.009 | 0.11 | 1 | 0.74 | -0.028 / 0.02 |
| % Correct choices in the distracting task | 0.019 | 0.012 | 2.59 | 1 | 0.107 | -0.013 / 0.052 |
| **Branching (right) *stimuli position (initial)** | **-0.127** | **0.034** | **6.33** | **1** | **0.01** | **-0.226/ -0.024** |
| **Kind of stimuli (spatial) *stimuli position (initial)** | **0.302** | **0.047** | **23.94** | **2** | **<0.001** | **0.16 / 0.426** |
| **Kind of stimuli (word) *stimuli position (initial)** | **-0.115** | **0.039** | **23.94** | **2** | **<0.001** | **-0.233 / -0.012** |

**Table S3.** Results for the STM Model. In bold, significant results. For details on the test categories, please see the text. In case interactions were tested, the main effects were also included in the model, but are not listed below.

| **Test Category** | **Estimate** | **SE** | **Chi-square** | **df** | ***p*** | **CI (95%) of the reduced model** |
| --- | --- | --- | --- | --- | --- | --- |
| Intercept | 0.099 | 0.109 | - | - | - | -0.187/0.412 |
| Sex | 0.016 | 0.028 | 0.33 | 1 | 0.56 | -0.060 / 0.091 |
| Age | -0.008 | 0.023 | 0.1 | 1 | 0.75 | -0.074 / 0.054 |
| Number of siblings | -0.011 | 0.019 | 0.37 | 1 | 0.54 | -0.063 / 0.037 |
| Residence | -0.06 | 0.051 | 1.19 | 1 | 0.27 | -0.205 / 0.087 |
| **Education** | **0.074** | **0.024** | **6.71** | **1** | **0.01** | **0.007 / 0.140** |
| Occupation 1  (Occupation 0 as reference) | 0.008 | 0.095 | 0.88 | 5 | 0.97 | -0.266 / 0.282 |
| Occupation 2 | 0.007 | 0.091 | 0.88 | 5 | 0.97 | -0.253 / 0.260 |
| Occupation 3 | -0.003 | 0.085 | 0.88 | 5 | 0.97 | -0.242 / 0.231 |
| Occupation 4 | -0.046 | 0.106 | 0.88 | 5 | 0.97 | -0.357 / 0.237 |
| Occupation 5 | -0.038 | 0.064 | 0.88 | 5 | 0.97 | -0.222 / 0.137 |
| Centered income | -0.048 | 0.044 | 1.12 | 1 | 0.28 | -0.176 / 0.073 |
| **Average national income** | **0.209** | **0.055** | **9.53** | **1** | **0.002** | **0.061 / 0.368** |
| Knowledge of another language 1 | 0.205 | 0.088 | 5.06 | 2 | 0.08 | -0.057 / 0.473 |
| Knowledge of another language 2 | 0.158 | 0.094 | 5.06 | 2 | 0.08 | -0.094 / 0.441 |
| Number of stimuli | 0.086 | 0.039 | 3.75 | 1 | 0.052 | -0.025 / 0.199 |
| Trial number | -0.0008 | 0.006 | 0.02 | 1 | 0.88 | -0.018 / 0.016 |
| Branching (right) | 0.191 | 0.112 | 2.22 | 1 | 0.14 | -0.128 / 0.506 |
| **Kind of stimuli (spatial)*stimuli position (initial)** | **0.361** | **0.0308** | **27.42** | **2** | **<0.001** | **0.273 / 0.449** |
| **Kind of stimuli (word) *stimuli position (initial)** | **-0.176** | **0.1** | **27.42** | **2** | **<0.001** | **-0.455 / 0.097** |

**6. References**

1. Lewis, M. P., Simons, G. F. & Fennig, C. D. *Ethnologue: languages of the world 19th edn. SIL International* (2016), (Available online at http://www.ethnologue.com).
2. Dryer, M. S. & Haspelmath, M. (eds) *The World Atlas of Language Structures Online* (Max Planck Institute for Evolutionary Anthropology, 2013), (Available online at http://wals.info).
3. World Bank. World development Indicators, 2015 (2015), (Available online at <http://data.worldbank.org/data-catalog/world-development-indicators>).
4. Unsworth, N., Heitz, R. P., Schrock, J. C. & Engle, R. W. An automated version of the operation span task. *Behav. Res. Meth.* **37***,* 498-505 (2005).
5. Unsworth, N., Fukuda, K., Awh, E. & Vogel, E. K. Working memory and fluid intelligence: capacity, attention control, and secondary memory retrieval. *Cogn. Psychol.* **71***,* 1-26 (2014).
6. Bowerman, M. in *Language and space* (eds Bloom, P., Peterson, M. A., Nadel, L., Garrett, M. F.) 385-436 (MIT Press, Cambridge, 1996).
7. Majid, A., Boster, J. S., Bowerman, M. The cross-linguistic categorization of everyday events: a study of cutting and breaking. *Cognition* **109**, 235-250 (2008).
8. Baayen, R. H., Davidson, D. J., Bates, D. M. Mixed-effects modeling with crossed random effects for subjects and items. *J. Mem.Lang.* **59***,* 390-412 (2005).
9. Bates, D. M. lme4: Mixed-effects modeling with R. Available online at: http://lme4. r-forge. r-project. org/book (2010).
10. Forstmeier, W. & Schielzeth, H. Cryptic multiple hypotheses testing in linear models: overestimated effect sizes and the winner's curse. *Behav. Ecol.Sociobiol.* **65***,* 47-55 (2011).
11. Barr, D. J., Levy, R., Scheepers, C. & Tily, H. J. Random effects structure for confirmatory hypothesis testing: keep it maximal. *J. Mem. Lang.* **68**, 255-278 (2013).
12. Field, A. *Discovering statistics using SPSS*. (Sage Publications, London, 2005).
